# Assessment of Potentially Inappropriate Prescribing of Opioid Analgesics Requiring Prior Opioid Tolerance

**DOI:** 10.1001/jamanetworkopen.2020.2875

**Published:** 2020-04-15

**Authors:** Molly Moore Jeffery, Christine E. Chaisson, Christopher Hane, Louis Rumanes, Jamie Tucker, Lillian Hang, Rozalina McCoy, Catherine L. Chen, Mark C. Bicket, W. Michael Hooten, Marc Larochelle, William C. Becker, Cynthia Kornegay, Judith A. Racoosin, Darshak Sanghavi

**Affiliations:** 1 Division of Health Care Policy Research, Mayo Clinic, Rochester, Minnesota; 2 OptumLabs, Cambridge, Massachusetts; 3 Robert D. and Patricia E. Kern Center for the Science of Health Care Delivery, Rochester, Minnesota; 4 Division of Community Internal Medicine, Mayo Clinic, Rochester, Minnesota; 5 Department of Anesthesia and Perioperative Care, University of California, San Francisco; 6 Department of Anesthesiology and Critical Care Medicine, Johns Hopkins University School of Medicine, Baltimore, Maryland; 7 Center for Drug Safety and Effectiveness, Johns Hopkins Bloomberg School of Public Health, Baltimore, Maryland; 8 Division of Pain Medicine, Mayo Clinic, Rochester, Minnesota; 9 Section of General Internal Medicine, Boston University School of Medicine, Boston, Massachusetts; 10 VA Connecticut Healthcare System, West Haven; 11 Yale School of Medicine, New Haven, Connecticut; 12 Center for Drug Evaluation and Research, US Food and Drug Administration, Silver Spring, Maryland; 13 Previously with OptumLabs, Cambridge, Massachusetts; 14 UnitedHealthcare, Boston, Massachusetts

## Abstract

**Importance:**

Opioid-tolerant only (OTO) medications, such as transmucosal immediate-release fentanyl products and certain extended-release opioid analgesics, require prior opioid tolerance for safe use, as patients without tolerance may be at increased risk of overdose. Studies using insurance claims have found that many patients initiating these medications do not appear to be opioid tolerant.

**Objectives:**

To measure prevalence of opioid tolerance in patients initiating OTO medications and to determine whether linked electronic health record (EHR) data contribute evidence of opioid tolerance not found in insurance claims data.

**Design, Setting, and Participants:**

This retrospective cohort study used a national database of deidentified longitudinal health information, including medical and pharmacy claims, insurance enrollment, and EHR data, from January 1, 2007, to December 31, 2016. Data included 131 756 US residents with at least 183 days of continuous enrollment in commercial or Medicare Advantage insurance (including medical and pharmacy benefits) who had received an OTO medication and who had no inpatient stays in the 30 days prior to starting an OTO medication; of these, 20 044 individuals had linked EHR data within the prior 183 days. Data were analyzed from July 1, 2017, to August 31, 2018.

**Exposures:**

Initiating an OTO medication.

**Main Outcomes and Measures:**

Prior opioid tolerance demonstrated through pharmacy fills or EHR data on prescriptions written.

**Results:**

Among 153 385 OTO use episodes identified, 89 029 (58.0%) occurred among women, 62 900 (41.0%) occurred among patients with Medicare Advantage insurance, 39 394 (25.7%) occurred in the Midwest, 17 366 (11.3%) occurred in the Northeast, 73 316 (47.8%) occurred in the South, and 23 309 (15.2%) occurred in the West. Less than half of use episodes (73 117 episodes [47.7%]) involved patients with evidence in claims data of opioid tolerance prior to initiating therapy with an OTO medication, including 31 392 of 101 676 episodes (30.9%) involving transdermal fentanyl, 1561 of 2440 episodes (64.0%) involving transmucosal fentanyl, 36 596 of 43 559 episodes (84.0%) involving extended-release oxycodone, and 3568 of 5710 episodes (62.5%) involving extended-release hydromorphone. Among 20 044 OTO use episodes with linked EHR and claims data, less than 1% of OTO episodes identified in claims had evidence of opioid tolerance in structured EHR data that was not present in claims data (108 episodes [0.5%]). After limiting the sample to OTO episodes identified in claims with a matching OTO prescription within 14 days in the structured EHR data, only 40 of 939 episodes (4.0%) occurred among patients with evidence of tolerance that was not present in claims data.

**Conclusions and Relevance:**

This cohort study found that most patients initiating OTO medications did not have evidence of prior opioid tolerance, suggesting they were at increased risk of opioid-related harms, including fatal overdose. Data from EHRs did not contribute substantial additional evidence of opioid tolerance beyond the data found in prescription claims. Future research is needed to understand the clinical rationale behind these observed prescribing patterns and to quantify the risk of harm to patients associated with potentially inappropriate prescribing.

## Key Points

**Question:**

What proportion of patients are opioid-tolerant when starting opioids that require prior tolerance for safe use?

**Findings:**

In this cohort study including 153 385 use episodes of opioid analgesics labeled only for use in people who are opioid-tolerant, more than half of patients who received these medications showed no evidence of prior opioid tolerance in health insurance claims or electronic health records.

**Meaning:**

These findings suggest that many patients may be at risk of potentially serious adverse events owing to widespread use of certain extended-release opioids without prior opioid tolerance.

## Teaser

This cohort study examines the prevalence of prescribing opioid analgesics that require prior tolerance for safe use to patients with no evidence of prior tolerance in insurance claims data or electronic health records.

## Introduction

For people who experience daily, around-the-clock pain, extended-release opioids may offer dosing convenience^1^ compared with immediate-release opioids, but they are associated with substantial risk of severe respiratory depression^2^ and overdose.^3,4^ Moreover, some extended-release opioid analgesics are formulated at strengths that should be reserved for patients with prior opioid tolerance.

Like several extended-release opioids, transmucosal immediate-release fentanyl (TIRF) products are labeled only for use in people who are opioid-tolerant. Transmucosal immediate-release fentanyl products are approved to treat breakthrough pain in adults with cancer who are already taking opioid analgesics around the clock. These drugs are highly potent and unsafe for patients who are not opioid-tolerant. Little is known about the extent to which patients initiating therapy with TIRFs show evidence of prior opioid tolerance.

Previously, Willy et al^5^ found that 44% of people enrolled in fee-for-service Medicare had no evidence of prior opioid tolerance when initiating high-dose extended-release oxycodone. Larochelle et al,^6^ reported that large proportions of people with commercial insurance had no evidence of prior tolerance when starting high-dose extended-release oxycodone (46%), extended-release hydromorphone (46%), and fentanyl transdermal systems (60%). A Canadian study of all transdermal fentanyl prescriptions in 12 years of data from Manitoba found similarly high rates of use in patients with no evidence of tolerance: 74.1% of prescribing episodes lacked evidence of prior tolerance.^7^ However, because these analyses relied on claims data, some evidence of prior opioid tolerance may not have been ascertained. Claims data may fail to capture prescriptions paid for in cash or with a different insurance policy (eg, for dual-enrollees in Medicare and Medicaid). Adding data from electronic health records (EHRs) could more accurately reflect the true proportion of patients receiving an extended-release opioid analgesic or TIRF product without prior opioid tolerance.

Linking claims and EHR data addresses some of the weaknesses of each data source used on its own. Claims data have a clear population from which health care use is drawn, defined by administrative records of people enrolled in health insurance coverage; procedures, supplies, and services covered by the insurance policy are likely to be captured by an insurance claim.^8^ However, nonbillable components of an individual’s health status or health care service use, medications filled with a cash payment, or a clinician’s unstructured clinical notes will not be captured by claims data.

Data from EHRs can provide these missing elements but are often confined to a specific health care system, such as a clinic or hospital. While EHR data may provide detailed clinical insights regardless of reimbursement, data on care received outside of these specific health care systems are likely to be absent.^9,10^ Data from EHRs capture only whether a prescription was written, not whether it was filled.

In this study, we used claims and linked EHR data to assess prior opioid tolerance in patients initiating therapy with certain extended-release opioid analgesics or TIRF products that require tolerance for safe use. We hypothesized that combining EHR and claims data would reduce the proportion of patients initiating opioid-tolerant-only (OTO) opioid regimens who were classified as nontolerant. We sought to reproduce prior claims-based analyses of opioid tolerance in a large sample of individuals enrolled in commercial insurance and Medicare Advantage, include TIRF products as well as the extended-release opioid analgesics previously studied, and extend the analyses by linking claims data to EHR data from structured fields and from unstructured clinical notes summarized using natural language processing techniques to determine whether any additional evidence of opioid tolerance could be detected from EHR data beyond that found in claims data.

## Methods

### Data Sources

This cohort study used data from the OptumLabs Data Warehouse (OLDW), a large health care database comprised of both insurance claims data and EHR data, supplemented with information from the full text of unstructured clinical notes. The insurance claims data are from a large, nationwide, commercial payer, and the EHR data are derived from a large network of hospitals, clinicals, and other health care organizations throughout the US. The databases are linked by unique identifiers shared across both data sets. In total, OLDW includes more than 100 million individuals. This retrospective study used preexisting, deidentified data; therefore, it did not require institutional review board approval or informed consent per 45 CFR 46.102. This study is reported following the Reporting of studies Conducted using Observational Routinely-collected Data (RECORD) reporting guidelines.

Three types of data were used in this study: insurance claims, structured EHR data, and unstructured clinical notes (ie, free-text EHR data). Key elements captured by insurance claims data include individual level characteristics (eg, sex, year of birth, geography), insurance type (eg, commercial or Medicare Advantage), enrollment periods, and health care use, including inpatient stays and opioid analgesic prescription fills. Structured EHR data are collected by clinicians at the point of care and include encounter details, such as contact dates and types (eg, telephone, emergency, outpatient), clinical procedures, diagnoses (including patient concerns); clinical observations (eg, blood pressures, pulse rates, respiratory rates); and treatment details, such as prescriptions written and medications administered. Unstructured clinical notes are data collected in the EHR that have no text entry restrictions. They may include dictated or typed notes from clinicians or automated text generated by the EHR system. To minimize the risk of identifying individual patients, the full text of clinical notes is not available to OLDW researchers. Notes data were accessed in a deidentified format with specific terms, sentiments (ie, opinions, feelings, or mood), and dates recorded.

### Cohorts

Three cohorts were used in this study: one created using only claims data, a second using structured EHR data linked to claims, and a third using a subset of the structured EHR data plus unstructured clinical notes linked to claims. The goal in all 3 cohorts was to select patients initiating use of one of the medications included in our analysis between January 1, 2007, and December 31, 2016. These medications include high-dose (ie, estimated cumulative daily dose >80 mg or dose unit of 60 or 80 mg) extended-release oxycodone, and all doses of extended-release hydromorphone, fentanyl transdermal systems (ie, fentanyl patches), and TIRF products. We refer to these medications as OTO medications because all are labeled only for use in people who are opioid-tolerant. We identified new episodes of OTO medication use, requiring a 183-day period with no use of that drug prior to the first day of the first prescription in the episode; because primary tolerance is measured at the time of the first prescription, an episode ends when the first prescription runs out (fill date + days supplied – 1 day). Inclusion criteria are summarized in Table 1, with further detail and cohort flow tables available in the eAppendix and eTables 1-4 in the Supplement.

**Table 1.** Selection Criteria and Key Definitions

Cohort	Inclusion criteria	Exclusion criteria	Opioid tolerance
Claims data only	Pharmacy claim for an OTO medication^a^ between January 1, 2007, and December 31, 2016; continuously enrolled in both medical and pharmacy coverage plans for at least 183 d prior to an OTO pharmacy claim; no evidence of OTO use of the same type in the 183 d prior to the OTO episode	No opioid poisoning diagnosis within the 183 d prior an OTO episode; no inpatient confinement in medical claims within the 30 d prior to an OTO episode; nonmissing age, sex, insurance type, and region	Evidence of total daily opioid dose ≥30 mg of oxycodone equivalents on each day of the 7 d prior to OTO episode, exclusive of start date
Structured EHR data only	Prescription order or medication administration for an OTO medication^a^ between July 1, 2007, and December 31, 2016; only prescription order and medication administration associated with a known, or direct, OTO national drug code were included; any evidence of EHR activity within 183 d prior to an OTO prescription order or administration; no evidence of OTO use of the same type in the 183 d prior to the OTO episode	No opioid poisoning diagnosis within the 183 d prior an OTO episode; no inpatient confinement in EHR data within the 30 d prior to an OTO episode; nonmissing age, sex, insurance type, and region	Evidence of total daily opioid dose ≥30 mg of oxycodone equivalents on each day of the 7 d prior to OTO episode, exclusive of start date
Unstructured clinical notes and limited structured EHR data	Prescription order for an OTO medication^b^ between July 1, 2007, and December 31, 2016; had any unstructured clinical notes within 30 d prior to an OTO prescription	None	Not possible with the data available to determine opioid tolerance; instead, we report any observed opioid exposure in the prior 30 d, including medications administered in a hospital

Abbreviations: EHR, electronic health record; OTO, opioid-tolerant only.

^a^ Defined as estimated cumulative daily dose of oxycodone greater than 80 mg or dose unit of 60 or 80 mg, any dose of extended-release hydromorphone, any dose of fentanyl transdermal system (fentanyl patch), or any dose of any transmucosal fentanyl product.

^b^ Defined as any dose of extended-release oxycodone, extended-release hydromorphone, fentanyl transdermal system (fentanyl patch), or any transmucosal fentanyl product. Extended release oxycodone of any dose was considered an OTO for this analysis because medication doses were not consistently observed.

### Outcomes

The primary outcome was opioid tolerance at the beginning of OTO episodes. We defined opioid tolerance based on the labeling requirements for the medications: at least 30 mg oxycodone equivalents on each of 7 days prior to the start of the OTO episode (Table 1). Sensitivity analyses used 3 additional opioid tolerance definitions from Larochelle et al^6^ that are less stringent than the primary tolerance definition (eAppendix in the Supplement). We also report opioid tolerance rates by dosage strength for transdermal fentanyl.

### Statistical Analysis

We used claims data to estimate the prevalence of opioid tolerance at the beginning of OTO episodes; we stratified the analysis by OTO medication, insurance type, year, and medication strength (transdermal fentanyl only). To determine whether structured EHR data provided additional evidence of opioid tolerance beyond that available in claims data, we first assessed the subset of patients with both structured EHR and claims data. We selected OTO episodes from the claims data analysis for which we had any evidence of structured EHR use in the prior 183 days. We then divided the episodes into 4 groups in a 2-by-2 table: evidence of tolerance in claims data (yes or no) vs evidence of tolerance in structured EHR data (yes or no). The goal was to determine the proportion of episodes with no evidence of prior opioid tolerance in claims data but with evidence of prior opioid tolerance in the structured EHR data. These potential cases of opioid tolerance would be missed in a claims-only analysis.

We repeated the analysis comparing evidence of tolerance in claims and structured EHR data in a smaller cohort for whom the initial OTO prescription was recorded in both claims data as a prescription fill and structured EHR data as a written prescription; in these cases, we hypothesized that any prior prescriptions that could demonstrate tolerance might be more likely to be present in the EHR for this cohort because there was a record of a prescribed opioid in the EHR (ie, the initial OTO prescription). To account for a possible lag between when the prescription was written and when it was filled, we allowed a difference of 14 days between the date in claims data and the date in structured EHR data.

Finally, we repeated the analysis comparing evidence of tolerance in claims data, structured EHR data, and unstructured notes. In this group, we required the note with the OTO prescription to match within 7 days of the claims fill date.

Unstructured clinical notes were analyzed using natural language processing (NLP) techniques to summarize information in unstructured EHR fields that might provide evidence of prior opioid tolerance. We present a brief description of the approach here, with more detail in the eAppendix and eTables 8-23 in the Supplement.

We combined all notes from the 30 days before each OTO episode into a single document per episode. Standard techniques were used to clean clinical notes, including stripping punctuation, proper names, very common words (appearing in ≥80% of notes), and rare words (appearing in ≤1% of notes). The goal was to identify terms that were specific and important—that is, they appeared in relatively few documents (ie, they were specific to those documents) but frequently in those documents (ie, when they appeared in a document, they represented an important aspect of the document).

Notes were further filtered to remove repetition from cut-and-paste notes and common template language, such as discharge instructions and templated review-of-systems text. A resultant matrix of more than 12 000 words and phrases was analyzed using a nonnegative matrix factorization model,^11^ beginning with 100 topics. A vector-space model was used to classify each document according to the frequency with which terms appeared in each document.

Following this step, members of a technical expert panel (including R.M., C.L.C., M.C.B., W.M.H., M.L., and W.C.B.) reviewed the topics and created a clinical classifier to interpret the underlying concept. Topics with no obvious interpretation were dropped from the final model. The technical expert panel included physicians and researchers with expertise in pain medicine, addiction medicine, critical care, primary care, psychiatry, and data science. We also consulted the technical expert panel to understand potential reasons a physician might prescribe an OTO medication to a patient who is not opioid-tolerant.

Analyses were completed using SAS statistical software version 9.4 (SAS Institute); Python toolkits and libraries, including *Natural Language Toolkit*, *NumPy*, *pandas*, and *scikit-learn*; and R statistical software version 3.4 (R Project for Statistical Computing). Data were analyzed from July 1, 2017, to August 31, 2018.

## Results

### Claims Analysis

From 2007 to 2016, we identified 153 385 OTO use episodes among 131 756 individuals. Among 153 385 OTO use episodes, 89 029 (58.0%) were among women, 62 900 (41.0%) were among patients with Medicare Advantage insurance, 39 394 (25.7%) occurred in the Midwest, 17 366 (11.3%) occurred in the Northeast, 73 316 (47.8%) occurred in the South, and 23 309 (15.2%) occurred in the West. The most common OTO medication was transdermal fentanyl (101 676 use episodes [66.3%], followed by high-dose extended-release oxycodone (43 559 use episodes [28.4%]), extended-release hydromorphone (5710 use episodes [3.7%]), and TIRFs (2440 use episodes [1.6%]) (Table 2). Most use episodes occurred in people aged 45 and older (125 053 episodes [81.5%]). Less than half of use episodes (73 117 episodes [47.7%]) occurred among patients with evidence in claims data of opioid tolerance prior to initiating therapy. The highest prevalence of prior opioid tolerance was seen for extended-release oxycodone (36 596 of 43 559 use episodes [84.0%]), followed by TIRFs (1561 of 2440 use episodes [64.0%]) and extended release hydromorphone (3568 of 5710 use episodes [62.5%]); the lowest prevalence of prior opioid tolerance was seen for transdermal fentanyl (31 392 of 101 676 use episodes [30.9%]) (Table 2). A cohort flow table is provided in eTable 1 in the Supplement.

**Table 2.** Episode Descriptive Statistics Derived From Claims Data

	No. (%)^a^				
	Extended-release		Fentanyl transdermal system (n = 101 676	TIRF (n = 2440)	Overall (N = 153 385)
	High-dose oxycodone (n = 43 559)^b^	Hydromorphone (n = 5710)			
Evidence of prior opioid tolerance	36 596 (84.0)	3568 (62.5)	31 392 (30.9)	1561 (64.0)	73 117 (47.7)
Age category, y					
0-17	>21 (>0.05)	<11 (<1.0)	124 (0.1)	<11 (<1.0)	167 (0.1)
18-24	<753 (<1.7)	>42 (<1.0)	846 (0.8)	>22 (<1.0)	1663 (1.1)
25-34	3611 (8.3)	402 (7.0)	4171 (4.1)	160 (6.6)	8344 (5.4)
35-44	7083 (16.3)	909 (15.9)	9760 (9.6)	406 (16.6)	18 158 (11.8)
45-54	12 334 (28.3)	1656 (29.0)	18 787 (18.5)	752 (30.8)	33 529 (21.9)
55-64	11 645 (26.7)	1692 (29.6)	23 133 (22.8)	722 (29.6)	37 192 (24.2)
65-74	5225 (12.0)	728 (12.7)	18 742 (18.4)	275 (11.3)	24 970 (16.3)
>75	2887 (6.6)	270 (4.7)	26 113 (25.7)	92 (3.8)	29 362 (19.1)
Women	21 372 (49.1)	3278 (57.4)	62 948 (61.9)	1431 (58.6)	89 029 (58.0)
Insurance type					
Medicare Advantage	12 596 (28.9)	2512 (44.0)	47 257 (46.5)	535 (21.9)	62 900 (41.0)
Census region					
Midwest	10 138 (23.3)	1026 (18.0)	27 905 (27.4)	325 (13.3)	39 394 (25.7)
Northeast	4326 (9.9)	536 (9.4)	12 223 (12.0)	281 (11.5)	17 366 (11.3)
South	20 822 (47.8)	3488 (61.1)	47 650 (46.9)	1356 (55.6)	73 316 (47.8)
West	8273 (19.0)	660 (11.6)	13 898 (13.7)	478 (19.6)	23 309 (15.2)

Abbreviations: TIRF, transmucosal fentanyl.

^a^ Cells not reported precisely are censored to prevent display of any cell size of fewer than 11 episodes.

^b^ Defined as oxycodone with an estimated cumulative daily dose greater than 80 mg or dose unit of 60 or 80 mg.

Generally, prevalence of prior tolerance was similar in the commercial and Medicare Advantage groups except for transdermal fentanyl use episodes in which patients with Medicare Advantage had a substantially lower prevalence of prior tolerance than patients with commercial insurance (12 090 episodes [25.6%] vs 19 302 episodes [35.5%]) (eFigure 1 and eFigure 2 in the Supplement). When tolerance prevalence was stratified by age, patients 75 years and older were the least likely to show evidence of opioid tolerance when initiating OTOs, with only of 6240 of 29 362 episodes (21.3%) among patients 75 years and older occurring with evidence of prior tolerance; this was particularly evident with transdermal fentanyl, for which only 3601 of 26 113 episodes (13.8%) among patients 75 years and older occurred with evidence of opioid tolerance, compared with 4309 of 9760 use episodes (44.1%) among patients aged 35 to 44 years occurring among patients with evidence of opioid tolerance (eFigure 3 in the Supplement).

Between 2007 and 2016 evidence of tolerance increased for patients who received extended-release oxycodone (change, 12.2 [95% CI, 10.6 to 13.9] percentage points), transdermal fentanyl (change, 3.5 [95% CI, 2.2 to 4.7] percentage points), and TIRFs (change, 18.4 [95% CI, 7.7 to 29.0] percentage points). The prevalence of prior tolerance for extended-release hydromorphone between 2010, when the drug first appeared in the OLDW database, and 2016 did not change significantly (change, 8.5 [95% CI, –2.9 to 20.0] percentage points). Complete details on prevalence of tolerance by year are presented in eFigures 4, 5, and 6 and eTable 7 in the Supplement.

Because the evidence of prior tolerance for newly prescribed transdermal fentanyl was particularly low, we calculated tolerance prevalence stratified by initial dosage strength and insurance group. At each dosage strength, patients with commercial insurance were more likely to show prior tolerance than patients with Medicare Advantage, and prior tolerance rates increased with higher dosage strengths (Figure; eTable 24 in the Supplement). Even at the highest dosage strength examined (100 μg/h), only half of patients with commercial insurance (889 of 1715 patients [51.8%]) and less than half of patients with Medicare Advantage (448 of 1005 patients [44.6%]) had evidence of prior tolerance.

**Figure.**
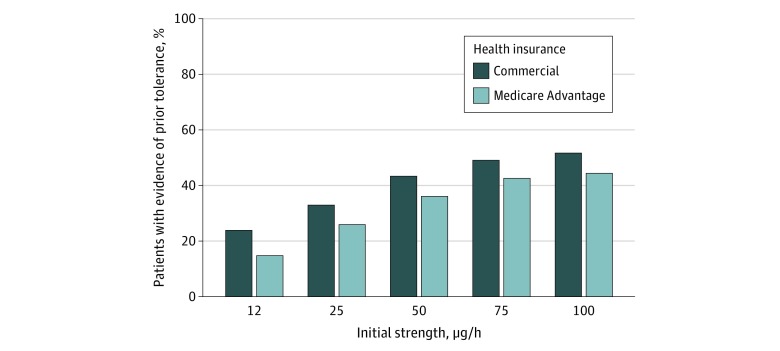
Evidence of Prior Opioid Tolerance Among Individuals Who Received Fentanyl Transdermal System Stratified by Initial Dose Strength

In a sensitivity analysis, we compared less stringent definitions of tolerance than the primary definition, which corresponds to the definition of opioid tolerance included in the US Food and Drug Administration–approved drug labels (>30 mg oxycodone equivalents in the prior 7 days). As expected, evidence of prior tolerance was higher with the less stringent definitions and differed by OTO medication type. For example, in the commercially insured population, prior tolerance for extended-release oxycodone varied from 25 589 of 30 963 use episodes (83.5%) using our primary study definition to 28 181 of 30 963 use episodes (91.0%) using the fourth and least restrictive sensitivity definition (rate ratio [RR], 1.09 [95% CI, 1.07 to 1.11]) (eFigure 2 in the Supplement). In comparison, transdermal fentanyl had the most variation, ranging from 19 302 of 54 419 use episodes (35.5%) among patients with evidence of tolerance using our primary definition to 39 344 of 54 419 use episodes (72.3%) among patients with evidence of tolerance using the least restrictive sensitivity definition in the commercially insured population (RR, 2.04 [95% CI, 2.00 to 2.07]); similar results were seen in the Medicare Advantage group (extended-release oxycodone: RR, 1.10 [95% CI, 1.07 to 1.13]; transdermal fentanyl: RR, 2.58 [95% CI, 2.58 to 2.64]) (eFigure 2, eFigure 3, eTable 5, and eTable 6 in the Supplement).

### Structured EHR Data Analysis

We identified 18 047 individuals contributing 20 044 OTO episodes that met the inclusion criteria for the analysis of EHR-derived structured data (13.8% of OTO episodes identified in claims between July 1, 2007, and December 31, 2016). The goal of this analysis was to identify additional evidence of prior opioid tolerance not captured in claims data. A detailed cohort definition is provided in eTable 2 in the Supplement.

Structured EHR data contributed minimal additional evidence of prior tolerance (Table 3). Less than 1% of OTO episodes identified in claims had evidence of opioid tolerance in structured EHR data that was not present in claims data (108 episodes [0.5%]). This evidence was contributed by records of opioid analgesic prescriptions written in the EHR data that did not appear as prescription fills in the claims data. These prescriptions may not have been filled, or they may have been filled but paid for with cash or by another source of insurance coverage.

**Table 3.** Evidence of Opioid Tolerance Added by Structured EHR Data

Evidence of primary opioid tolerance in structured EHR data	Evidence of primary opioid tolerance in claims data, No. (%)					
	OTO episode ascertained using claims data			OTO episode ascertained matching claims data to EHR data		
	Yes	No	Total	Yes	No	Total
Yes	520 (2.5)	108 (0.5)	628 (3.1)	271 (28.8)	40 (4.3)	311 (33.1)
No	8725 (43.5)	10 691 (53.3)	19 416 (96.9)	120 (12.8)	508 (54.1)	628 (66.9)
Total	9245 (46.1)	10 799 (53.8)	20 044	391 (41.6)	548 (58.4)	939

Abbreviation: EHR, electronic health record; OTO, opioid-tolerant only.

When we further limited the sample to 939 OTO episodes identified in claims with a matching OTO prescription within 14 days in the structured EHR data, we again found little additional evidence of tolerance contributed by structured EHR data (Table 3). Only 40 of 939 episodes (4.0%) had evidence of tolerance in the structured EHR data that was not present in claims data.

### EHR Unstructured Clinical Notes Analysis

Finally, to search for evidence of prior tolerance not present in either claims or structured EHR data, we used natural language processing techniques to summarize the clinical notes from the 30 days preceding an OTO episode (Table 1). We identified 46 525 episodes eligible for this analysis (eTable 14 and eTable 15 in the Supplement).

Topics were reviewed by the technical expert panel and given a descriptive name. Twenty-six topics that had too little total weight (ie, total weight <0.01) and 8 topics that had too many words (ie, exceeding the 90th percentile [279 words]) were dropped from the model, leaving 66 topics (eTable 19 in the Supplement).

After reviewing the topics with our technical expert panel, we suspected the topics were unlikely to identify additional evidence of tolerance because most topics were related to specific illnesses and health services unrelated to opioid tolerance (eg, laboratory tests, lower extremity ailments). We asked the technical expert panel to help us understand why a patient who was not opioid-tolerant might receive an OTO product. From that discussion, we identified additional terms, which we called *whitelist terms*, that might suggest a reason these patients received an OTO medication (Box; eTables 8, 20, 21, 22, and 23 in the Supplement).

Box. **Reasons for OTO Prescriptions for Patients Without Opioid Tolerance**

**Patient-Related Reasons**

- Vomiting or gastrointestinal issues
- Swallowing issues
- Dementia or difficulty following dosing instructions
- Patient using many medications daily or attempt to fit opioid into existing medication regimen (eg, morning and evening)
- Patient distress or other treatments failing
- Sleep issues

**Clinician-Related Reasons**

- Residency or training
  - Clinician taught that extended-release opioids are better than immediate-release for some patients
  - Not enough training on pain management
  - Prescriber wants to avoid attending physician being called to manage pain overnight
- Lack of familiarity with product labeling requirements

Terms and topics from the unstructured clinical notes were linked to claims data for 249 episodes in which the initial OTO prescription was recorded in the claims data as a prescription fill and in the structured EHR data as a written prescription along with a clinical note within 7 days of the claim. To determine whether textual analysis provided evidence of opioid tolerance, we looked for topics and terms that seemed to represent opioid tolerance or conversely, prescribing for individuals who were not opioid-tolerant. None of the topics identified by natural language processing analysis directly represented opioid use in individuals who were not opioid-tolerant.

We also used bivariate and multivariate analysis to assess the associations of terms or topics with opioid tolerance at the start of the episode. The prespecified analytic plan was to use 5-fold cross-validation to solve a lasso logistic regression model. However, the lack of clearly written reasons for prescribing and low term counts made this approach unfruitful.

To address the infrequency with which any 1 term was observed, we grouped the whitelist words developed by our technical expert panel to identify possible reasons for prescribing OTO medications to individuals who were not opioid-tolerant. The groups were patient request or affective components, vomiting or gastrointestinal issues, sleep issues, cognitive or functional deficit, addiction, or pain severity or lack of response or patient unable to take other drugs. We calculated unadjusted RRs assessing the relative risk of opioid nontolerance among patients whose notes contained any of the terms (exposed) vs those that did not (unexposed) (eTable 18 in the Supplement). None of the grouped topics had statistically significant differences in opioid nontolerance.

## Discussion

In this cohort study using a large linked database of commercial claims, structured EHR, and unstructured clinical notes data, we confirmed findings from prior claims-based studies^5,6^ demonstrating lack of prior opioid tolerance in a substantial proportion of patients initiating certain extended-release opioid analgesics that require prior opioid tolerance. Complete comparisons of this study’s findings with findings reported by Larochelle et al^6^ and Willy et al^5^ are presented in eTables 25-29 and eFigure 7 in the Supplement. We also found that a large proportion of patients initiating TIRF products did not have evidence of prior opioid tolerance in claims data.

We did not find substantial additional evidence of prior opioid tolerance when we supplemented claims data with structured EHR data and unstructured clinical notes. Prescriptions paid for with cash or an alternative source of insurance might explain some cases of apparent nontolerance when claims data were used alone. However, our findings suggest that prescription fills missing from claims data are unlikely to explain the low levels of opioid tolerance observed in claims analysis.

Although there was some improvement over time in prevalence of prior opioid tolerance among patients prescribed OTO medications, our findings suggest prescribers frequently use OTOs in patients who are not opioid-tolerant, which does not adhere to the labeling requirements for the products studied. Even for the highest dosage-strength TIRF (100 μg/h), the prevalence of prior tolerance was just 44.6% for patients with Medicare Advantage and 51.8% for patients with commercial insurance.

Our technical expert panel suggested possible reasons or justifications for use of OTO medications in people who are opioid nontolerant, as noted in the Box. These reasons included lack of knowledge of the need for opioid tolerance when starting OTO medications, as well as patient-related reasons for use of extended-release opioid analgesic medications. Many reasons were specific to issues addressed by fentanyl transdermal systems (eg, severe gastrointestinal problems that make oral consumption impossible and favor a transdermal approach for pain management). However, we were unable to assess what motivated clinicians prescribing OTO medications to individuals without evidence of opioid tolerance.

In a sensitivity analysis using 3 less restrictive definitions of tolerance, we observed variation by OTO type. Transdermal fentanyl had the most variation in the sensitivity analysis, increasing the rates of evidence of tolerance approximately 2-fold from the drug label definition to the least stringent definition, which only required 7 days with any non-0 opioid dose in the 30 days prior to the index OTO prescription. A prior study using commercial claims data and the same tolerance definitions similarly found much greater variability across the definitions in tolerance for individuals who used transdermal fentanyl than for individuals who used extended-release oxycodone.^6^ In part, this increased variability reflects the much lower rates of tolerance for transdermal fentanyl compared with the other drugs—there is more room for rates to increase. Beyond this mechanical explanation, the finding may reflect different patterns in prescribing of these drugs: extended-release oxycodone is available at lower non-OTO doses, making it likely that prescribers would start patients at 1 of these doses before escalating to an OTO dose. Use of extended-release hydromorphone and TIRFs was rare in this study, making it difficult to interpret these data; TIRF products are only indicated to treat breakthrough pain in cancer patients who are already on an opioid around the clock and would not be the first-line opioid. By contrast, transdermal fentanyl was the most common drug in our study and is available at a low dose, which may make it more likely to be started after minimal prior trials of other opioids.

### Limitations

Our study has several limitations. We were not able to review complete medical records to assess reasons for prescribing OTO medications, and topic modeling may not have captured all clinical justifications. Study analyses that used claims data included patients with commercial insurance and patients with Medicare Advantage; it is not known whether these results would be generalizable to other populations, including patients with Medicaid or patients who have no insurance. Although the data sources we used are very large, when we linked them and required evidence of the initial OTO prescription in claims and EHR data, our final sample was relatively small. We were able to ascertain only which medications were prescribed and dispensed to patients but not how much patients actually used nor when they used it. Both claims and EHR data are collected for purposes other than research and do not contain enough information to draw definitive conclusions about the appropriateness of individual patients’ medical care. Additionally, this study does not address the question of whether patients were harmed by potentially inappropriate use of OTO medications.

## Conclusions

This cohort study found that data from EHRs did not contribute substantial additional evidence of opioid tolerance to claims data. Missing prescription fills are unlikely to account for the large proportion of patients who are opioid nontolerant when initiating OTO medications. These patients may be at risk for opioid-related harms, including fatal overdose. Future research is needed to understand the clinical rationale behind these observed prescribing patterns and to quantify the risk of harm to patients associated with potentially inappropriate prescribing.

## Notes

Supplement. **eAppendix.** Supplementary Methods **eTable 1.** Claims Analysis Sample Cohort Flow **eTable 2.** Study Samples for Electronic Health Record Structured Fields Plus Claims Data Analyses **eTable 3.** Structured Electronic Health Record and Claims Data Analysis Study Sample Cohort Flow (Main Analysis) **eTable 4.** Structured Electronic Health Record and Claims Data Analysis Study Sample Cohort Flow (Secondary Analysis) **eFigure 1.** Proportion of Incident Opioid-Tolerant Only Episodes Meeting Criteria for Tolerance in Commercial Population **eFigure 2.** Proportion of Incident Opioid-Tolerant Only Episodes Meeting Criteria for Tolerance in Medicare Advantage Population **eTable 5.** Opioid Tolerance in Opioid-Tolerant Only Episodes Identified in Electronic Health Record Structured Fields (Main Analysis) **eTable 6.** Opioid Tolerance in Opioid-Tolerant Only Episodes Identified in Electronic Health Record Structured Fields (Secondary Analysis) **eFigure 3.** Opioid Tolerance Claims Analyses Stratified by Age and Opioid-Tolerant Only Medication **eTable 7.** Annual Trends of Evidence for Primary Opioid Tolerance by Opioid-Tolerant Only Type in OptumLabs Data Warehouse **eFigure 4.** Annual Trends in Evidence of Opioid Tolerance in Commercial Episodes in OptumLabs Data Warehouse **eFigure 5.** Annual Trends in Evidence of Opioid Tolerance in Medicare Advantage Episodes in OptumLabs Data Warehouse **eFigure 6.** Annual Trends in Evidence of Opioid Tolerance in Overall Episodes in OptumLabs Data Warehouse **eTable 8.** Sample of White List Words Generated by the Technical Expert Panel **eTable 9.** Example of Copy and Paste **eTable 10.** Single-Word Phrase Vector **eTable 11.** Multiword Phrase Vector **eTable 12.** Phrases to be Converted Into Vector Matrix **eTable 13.** Vector Matrix of Phrases in eTable 12 **eTable 14.** Clinical Notes Cohort **eTable 15.** Demographics of Clinical Notes Cohort **eTable 16.** Example of Topics Before Cleaning Generated From Clinical Notes **eTable 17.** Final Topics Matrix Cohort After Merging With Claims **eTable 18.** Risk of Nontolerance Among Patients With and Without Terms, With Both Claims and Free-Text Notes Electronic Medical Record Data **eTable 19.** List of 100 Topics from NMF Model **eTable 20.** Terms in Addiction Category **eTable 21.** Terms in Sleep Category **eTable 22.** Terms in Cognitive or Functional Deficit Category **eTable 23.** Terms in Patient Request or Affective Components Category **eTable 24.** Evidence of Tolerance Among Patients Receiving Transdermal Fentanyl Stratified by Initial Dose Strength and Insurance Group **eTable 25.** Distribution of Opioid-Tolerant Only Episodes by Medication Type in Larochelle et al and OptumLabs Data Warehouse **eTable 26.** Comparison of Age and Gender in Larochelle et al and OptumLabs Data Warehouse **eTable 27.** Comparison of Age and Gender in Willy et al and OptumLabs Data Warehouse **eTable 28.** Opioid Tolerance Rate by Age in Willy et al vs OptumLabs Data Warehouse **eTable 29.** Opioid Tolerance Rate by Age in Larochelle et al vs OptumLabs Data Warehouse **eFigure 7.** Comparison of Opioid-Tolerant Only Episodes Meeting Tolerance Definitions 1 to 4 in Larochelle et al and OptumLabs Data Warehouse **eTable 30.** Opioid Poisoning Diagnosis Codes **eReferences.**

## References

[1] Argoff CE, Silvershein DI A comparison of long- and short-acting opioids for the treatment of chronic noncancer pain: tailoring therapy to meet patient needs. Mayo Clin Proc. 2009;84(7):-. doi:10.1016/S0025-6196(11)60749-0 19567714 PMC2704132

[2] Zedler B, Xie L, Wang L, Development of a risk index for serious prescription opioid-induced respiratory depression or overdose in Veterans’ Health Administration patients. Pain Med. 2015;16(8):1566-1579. doi:10.1111/pme.12777 26077738 PMC4744747

[3] Miller M, Barber CW, Leatherman S, Prescription opioid duration of action and the risk of unintentional overdose among patients receiving opioid therapy. JAMA Intern Med. 2015;175(4):608-615. doi:10.1001/jamainternmed.2014.8071 25686208

[4] Park TW, Lin LA, Hosanagar A, Kogowski A, Paige K, Bohnert AS Understanding risk factors for opioid overdose in clinical populations to inform treatment and policy. J Addict Med. 2016;10(6):369-381. doi:10.1097/ADM.0000000000000245 27525471

[5] Willy ME, Graham DJ, Racoosin JA, Candidate metrics for evaluating the impact of prescriber education on the safe use of extended-release/long-acting (ER/LA) opioid analgesics. Pain Med. 2014;15(9):1558-1568. doi:10.1111/pme.12459 24828968

[6] Larochelle MR, Cocoros NM, Popovic J, Opioid tolerance and urine drug testing among initiates of extended-release or long-acting opioids in Food and Drug Administration’s Sentinel system. J Opioid Manag. 2017;13(5):315-327. doi:10.5055/jom.2017.0407 29199397

[7] Friesen KJ, Woelk C, Bugden S Safety of fentanyl initiation according to past opioid exposure among patients newly prescribed fentanyl patches. CMAJ. 2016;188(9):648-653. doi:10.1503/cmaj.150961 27044480 PMC4902689

[8] Lin KJ, Schneeweiss S Considerations for the analysis of longitudinal electronic health records linked to claims data to study the effectiveness and safety of drugs. Clin Pharmacol Ther. 2016;100(2):147-159. doi:10.1002/cpt.359 26916672

[9] Hersh WR, Weiner MG, Embi PJ, Caveats for the use of operational electronic health record data in comparative effectiveness research. Med Care. 2013;51(8)(suppl 3):S30-S37. doi:10.1097/MLR.0b013e31829b1dbd 23774517 PMC3748381

[10] Weng C, Appelbaum P, Hripcsak G, Using EHRs to integrate research with patient care: promises and challenges. J Am Med Inform Assoc. 2012;19(5):684-687. doi:10.1136/amiajnl-2012-000878 22542813 PMC3422845

[11] Lee DD, Seung HS Learning the parts of objects by non-negative matrix factorization. Nature. 1999;401(6755):788-791. doi:10.1038/44565 10548103

