# Supplementary material for: Assessment of Potentially Inappropriate Prescribing of Opioid Analgesics Requiring Prior Opioid Tolerance
Source: JAMA Netw Open. 2020 Apr 15;3(4):e202875. doi: 10.1001/jamanetworkopen.2020.2875 (PMC7160686; doi:10.1001/jamanetworkopen.2020.2875)
Supplement: Supplement. — eAppendix. Supplementary Methods eTable 1. Claims Analysis Sample Cohort Flow eTable 2. Study Samples for Electronic Health Record Structured Fields Plus Claims Data Analyses eTable 3. Structured Electronic Health Record and Claims Data Analysis Study Sample Cohort Flow (Main Analysis) eTable 4. Structured Electronic Health Record and Claims Data Analysis Study Sample Cohort Flow (Secondary Analysis) eFigure 1. Proportion of Incident Opioid-Tolerant Only Episodes Meeting Criteria for Tolerance in Commercial Population eFigure 2. Proportion of Incident Opioid-Tolerant Only Episodes Meeting Criteria for Tolerance in Medicare Advantage Population eTable 5. Opioid Tolerance in Opioid-Tolerant Only Episodes Identified in Electronic Health Record Structured Fields (Main Analysis) eTable 6. Opioid Tolerance in Opioid-Tolerant Only Episodes Identified in Electronic Health Record Structured Fields (Secondary Analysis) eFigure 3. Opioid Tolerance Claims Analyses Stratified by Age and Opioid-Tolerant Only Medication eTable 7. Annual Trends of Evidence for Primary Opioid Tolerance by Opioid-Tolerant Only Type in OptumLabs Data Warehouse eFigure 4. Annual Trends in Evidence of Opioid Tolerance in Commercial Episodes in OptumLabs Data Warehouse eFigure 5. Annual Trends in Evidence of Opioid Tolerance in Medicare Advantage Episodes in OptumLabs Data Warehouse eFigure 6. Annual Trends in Evidence of Opioid Tolerance in Overall Episodes in OptumLabs Data Warehouse eTable 8. Sample of White List Words Generated by the Technical Expert Panel eTable 9. Example of Copy and Paste eTable 10. Single-Word Phrase Vector eTable 11. Multiword Phrase Vector eTable 12. Phrases to be Converted Into Vector Matrix eTable 13. Vector Matrix of Phrases in eTable 12 eTable 14. Clinical Notes Cohort eTable 15. Demographics of Clinical Notes Cohort eTable 16. Example of Topics Before Cleaning Generated From Clinical Notes eTable 17. Final Topics Matrix Cohort After Merging With Claims eTable 18. Risk of [file jamanetwopen-3-e202875-s001.pdf]

Supplementary Online Content

Jeffery MM, Chaisson CE, Hane C, et al. Assessment of potentially inappropriate prescribing of opioid analgesics requiring prior opioid tolerance. *JAMA Netw Open*. 2020;3(4):202875. doi:10.1001/jamanetworkopen.2020.2875

eAppendix. Supplementary Methods
eTable 1. Claims Analysis Sample Cohort Flow
eTable 2. Study Samples for Electronic Health Record Structured Fields Plus Claims Data Analyses
eTable 3. Structured Electronic Health Record and Claims Data Analysis Study Sample Cohort Flow (Main Analysis)
eTable 4. Structured Electronic Health Record and Claims Data Analysis Study Sample Cohort Flow (Secondary Analysis)
eFigure 1. Proportion of Incident Opioid-Tolerant Only Episodes Meeting Criteria for Tolerance in Commercial Population
eFigure 2. Proportion of Incident Opioid-Tolerant Only Episodes Meeting Criteria for Tolerance in Medicare Advantage Population
eTable 5. Opioid Tolerance in Opioid-Tolerant Only Episodes Identified in Electronic Health Record Structured Fields (Main Analysis)
eTable 6. Opioid Tolerance in Opioid-Tolerant Only Episodes Identified in Electronic Health Record Structured Fields (Secondary Analysis)
eFigure 3. Opioid Tolerance Claims Analyses Stratified by Age and Opioid-Tolerant Only Medication
eTable 7. Annual Trends of Evidence for Primary Opioid Tolerance by Opioid-Tolerant Only Type in OptumLabs Data Warehouse
eFigure 4. Annual Trends in Evidence of Opioid Tolerance in Commercial Episodes in OptumLabs Data Warehouse
eFigure 5. Annual Trends in Evidence of Opioid Tolerance in Medicare Advantage Episodes in OptumLabs Data Warehouse
eFigure 6. Annual Trends in Evidence of Opioid Tolerance in Overall Episodes in OptumLabs Data Warehouse
eTable 8. Sample of White List Words Generated by the Technical Expert Panel
eTable 9. Example of Copy and Paste
eTable 10. Single-Word Phrase Vector
eTable 11. Multiword Phrase Vector
eTable 12. Phrases to be Converted Into Vector Matrix
eTable 13. Vector Matrix of Phrases in eTable 12
eTable 14. Clinical Notes Cohort
eTable 15. Demographics of Clinical Notes Cohort
eTable 16. Example of Topics Before Cleaning Generated From Clinical Notes
eTable 17. Final Topics Matrix Cohort After Merging With Claims
eTable 18. Risk of Nontolerance Among Patients With and Without Terms, With Both Claims and Free-Text Notes Electronic Medical Record Data
eTable 19. List of 100 Topics from NMF Model
eTable 20. Terms in Addiction Category
eTable 21. Terms in Sleep Category
eTable 22. Terms in Cognitive or Functional Deficit Category

© 2020 Jeffery MM et al. *JAMA Network Open.*

eTable 23. Terms in Patient Request or Affective Components Category
eTable 24. Evidence of Tolerance Among Patients Receiving Transdermal Fentanyl Stratified by Initial Dose Strength and Insurance Group
eTable 25. Distribution of Opioid-Tolerant Only Episodes by Medication Type in Larochelle et al and OptumLabs Data Warehouse
eTable 26. Comparison of Age and Gender in Larochelle et al and OptumLabs Data Warehouse
eTable 27. Comparison of Age and Gender in Willy et al and OptumLabs Data Warehouse
eTable 28. Opioid Tolerance Rate by Age in Willy et al vs OptumLabs Data Warehouse
eTable 29. Opioid Tolerance Rate by Age in Larochelle et al vs OptumLabs Data Warehouse
eFigure 7. Comparison of Opioid-Tolerant Only Episodes Meeting Tolerance Definitions 1 to 4 in Larochelle et al and OptumLabs Data Warehouse
eTable 30. Opioid Poisoning Diagnosis Codes
eReferences.

This supplementary material has been provided by the authors to give readers additional information about their work.

© 2020 Jeffery MM et al. *JAMA Network Open.*

eAppendix. Supplementary Methods

Description of OptumLabs Data Warehouse

OptumLabs® is an open, collaborative research and innovation center founded in 2013 as a partnership between Optum and Mayo Clinic with its core linked data assets in the OptumLabs Data Warehouse (OLDW). The database contains de-identified, longitudinal health information on enrollees and patients, representing a diverse mixture of ages, ethnicities and geographical regions across the United States. The claims data in OLDW includes medical and pharmacy claims, laboratory results and enrollment records for commercial and Medicare Advantage enrollees. The EHR-derived data includes a subset of EHR data that has been normalized and standardized into a single database.

Further information, including a list of peer-reviewed publications using OLDW, is available from the OptumLabs website: https://www.optumlabs.com/.

Claims data-only analysis

Inclusion criteria

Data from July 1, 2006 through December 31, 2016 were used in this analysis. This used the most recently available data in the OptumLabs Data Warehouse (OLDW) data stream at the time of data extraction. The time from July 1, 2006 and Dec 31, 2006 was used only to ascertain whether an opioid-tolerant-only (OTO) episode starting in the first six months of the study period

© 2020 Jeffery MM et al. *JAMA Network Open.*

represented incident use. A 183-day washout period with no use of opioids at dosages requiring prior tolerance was required after the end of any prior OTO episodes to define a new OTO episode.

Episode definition

OTO episodes were defined as follows:

For all opioids prescribed at doses requiring prior tolerance, an episode is defined as:

start date = fill date; and end date = [(fill date + days' supply) – 1].

For episodes of use of extended release oxycodone, the episode start date was defined as the first the date where cumulative daily dosage exceeded 80 mg or drug strength was equal to 60 mg or 80 mg. The episode end date was defined as the latest date where the cumulative daily dosage exceeded 80 mg or the runout of the claim of the 60 mg or 80 mg fill.

Assumptions Regarding Days' Supply: claims data analysis

Because prescription claims indicate only that medications were retrieved from the pharmacy, with no definitive information about patient behavior in taking these medications, the following assumptions were made:

- **Assumption 1:** The quantity of medication dispensed was consumed equally over the days' supplied from pharmacy claims
- **Assumption 2:** Patients began taking medications on the fill date.
- **Assumption 3:** The patient took all medication dispensed, leaving no excess supply.

© 2020 Jeffery MM et al. *JAMA Network Open.*

This analysis was conducted in the OLDW environment using claims data. The unit of observation was an OTO episode that met the following criteria:

- The enrollee had both medical and pharmacy benefits and was enrolled in a Medicare Advantage or commercial plan.
- Evidence of an OTO prescription between January 1, 2007 and December 31, 2016.
- 6 months (183 days) of continuous enrollment in medical and pharmacy benefits prior to and including date of the OTO prescription
- No evidence of an OTO prescription of the same type during the 183 days prior to OTO episode (that is, the episode is incident)
- Episodes (not individuals) were excluded for any of the following reasons:
    - Quantity or days' supplied on the qualifying claim was ≤0.

The enrollee had an opioid poisoning diagnosis in any position in the 183 days (washout period) prior to and including the start date of the episode. Opioid poisoning was defined as an opioid poisoning code in any diagnosis code position on any claim (See

- for code list).
- The enrollee had evidence of an inpatient confinement in the 30 days prior to and including the start date of the episode. This exclusion is applied because patients may be started on an opioid in the hospital without a record in claims; medication information during hospital stays is not complete in claims data, particularly for oral medications.

© 2020 Jeffery MM et al. *JAMA Network Open.*

- The enrollee has a missing or unknown age, gender, insurance type, or region.

eTable 1. Claims Analysis Sample Cohort Flow

	Episodes	Individuals
Evidence of new opioid-tolerant-only dose episodes	294,502	247,828
No opioid poisoning diagnosis 183 days prior to start of new episode	293,300	
Continuously enrolled in medical and pharmacy 183 days prior to start of new episode	194,126	
Episodes with known gender, age census region, and business line of individuals	193,536	
No inpatient stays 30 days prior to start of new episode	153,385	131,756

Structured Electronic Medical Recordand Claims data analysis

Description of Electronic Medical Recorddata

EHR data in OLDW is derived from dozens of healthcare provider organizations in US, with approximately 700 hospitals and 7,500 clinics, treating >64 million patients. The information available in EHR data is rich, including, but not limited to, test results, prescriptions written, and patient vital signs. However, unlike in claims data where we have a clearly defined group of people for whom we see claims for almost all significant health care events, there is no similar clearly defined denominator group in an EHR environment. When we see a period of time with no claims for a person with continuous enrollment, we can be relatively certain that person has received no major care. If we see that in EHR data, it could be the case that the person has received no care, or it could be that that the person has received care in a system on a different

© 2020 Jeffery MM et al. *JAMA Network Open.*

EHR. We have no way to determine which of these is true. This concept is referred to as "leakage." It is a major consideration in analysis of EHR data. Still, careful analysis of EHR data can yield important insight as long as the limitations are understood.

The EHR data in OLDW:

- Includes clinical data from patients of all insurance types as well as uninsured
- Covers several important patient activities with both structured data and free-text clinical notes, including:
  - Outpatient Office Visits
  - Consultation Reports
  - Operative (Procedure) Reports
  - Admission, Discharge Summaries
  - Nursing
  - Labs
  - Emergency Department
  - Pathology
  - Radiology
  - Cardiology

Structured data analyses:

In general, the analyses using EHR data followed the same procedures as the claims data analyses. There were two key exceptions, described here:

© 2020 Jeffery MM et al. *JAMA Network Open.*

1. The EHR data availability began in Jan. 1, 2007. As a result, the analyses including EHR data used a slightly shorter timeframe from July 1, 2007 through December 31, 2016 – six months shorter than the claims analysis. As in the claims analysis, the first six months of data were used only to ascertain whether study period OTO use was incident use.

2. In the electronic medical record data, missing National Drug Code (NDC codes) and accompanying dose information are frequently filled in with derived NDC codes based on common prescribing patterns. Derived NDC codes were not used to identify OTO episodes. Episode calculations otherwise followed the method used for claims.

Eligibility criteria:

- Evidence of any activity in the EHR database in the 6 months (183 days) prior to the OTO episode date. This evidence could consist of any kind of record in structured clinical data, including labs tests and need not have been related to opioid prescribing.

- A prescription record for an OTO medication that included 1) a non-derived NDC code for the OTO medication and 2) non-missing dose and duration

- For the secondary analysis, also required evidence of the OTO episode in both claims data and EHR data: the same OTO drug appearing as a pharmacy fill in the claims data and as a prescription in the EHR data within 14 days of each other

eTable 2. Study Samples for Electronic Health Record Structured Fields Plus Claims Data Analyses

Sample/subsample	Denominator	Numerators	Evaluation
Main analysis<br>• Individuals who have both	OTO episodes in Claims	• Evidence of opioid tolerance in EHR	Evaluate whether additional evidence of tolerance is provided by EHR

© 2020 Jeffery MM et al. *JAMA Network Open.*

claims and EHR data<br><br>• OTO captured in Claims<br><br>• Tolerance identified in EHR and claims	AND<br><br>EHR activity within 183 days to prior OTO	indicating tolerance<br><br>• Evidence of opioid tolerance in claims indicating tolerance	data in the OTO episodes that were identified in claims (regardless of whether OTO was also identified in EHR)
Secondary analysis<br><br>• Individuals who have both claims and EHR data concurrently<br><br>• OTO captured in both Claims and EHR<br><br>• Tolerance defined in EHR and claims	OTO episodes in Claims<br>AND<br>Matched OTO episodes in EHR (prescription for same OTO drug identified in the EHR data within 14 days of claims fill date)	• Evidence of opioid tolerance in EHR indicating tolerance<br><br>• Evidence of opioid tolerance in claims indicating tolerance	Evaluate whether additional evidence of tolerance is provided by EHR data in the OTO episodes that were identified in both claims and EHR

eTable 3. Structured Electronic Health Record and Claims Data Analysis Study Sample Cohort Flow (Main Analysis)

	N	%
OTO Episode identified in claims	145,416	100%
EHR data at any time	45,776	31%
EHR Data in the 183 days prior to OTO	20,044	14%

eTable 4. Structured Electronic Health Record and Claims Data Analysis Study Sample Cohort Flow (Secondary Analysis)

	N
OTO Episode identified in claims	145,416
EHR data at any time	45,776
EHR Data in the 183 days prior to OTO	20,044
Matching OTO type identified in EHR within 30 days	1,002
Matching OTO type identified in EHR within 14 days	939
For comparison: matching within 7 days did not change the sample substantially, so we report the 14 day match	
Matching OTO type identified in EHR within 7 days	*914*

Sensitivity analysis results: alternative definitions of opioid tolerance

- Tolerance definition 1 [used in manuscript]: Evidence of ≥30 mg of oxycodone equivalents on each day of the 7 days prior to OTO episode, exclusive of start date
- Tolerance definition 2: Evidence of at least 7 days of ≥30 mg of oxycodone equivalents in the 30 days prior to OTO episode, exclusive of start date. The seven days are not required to be consecutive.
- Tolerance definition 3: Evidence of >0 mg of oxycodone equivalents on each of the 7 days prior OTO episode, exclusive of start date
- Tolerance definition 4: Evidence of at least 7 days of >0 mg oxycodone equivalents in the 30 days prior to the OTO episode, exclusive of start date. The seven days are not required to be consecutive.

© 2020 Jeffery MM et al. *JAMA Network Open.*

Claims only analysis

eFigure 1. Proportion of Incident Opioid-Tolerant Only Episodes Meeting Criteria for Tolerance in Commercial Population

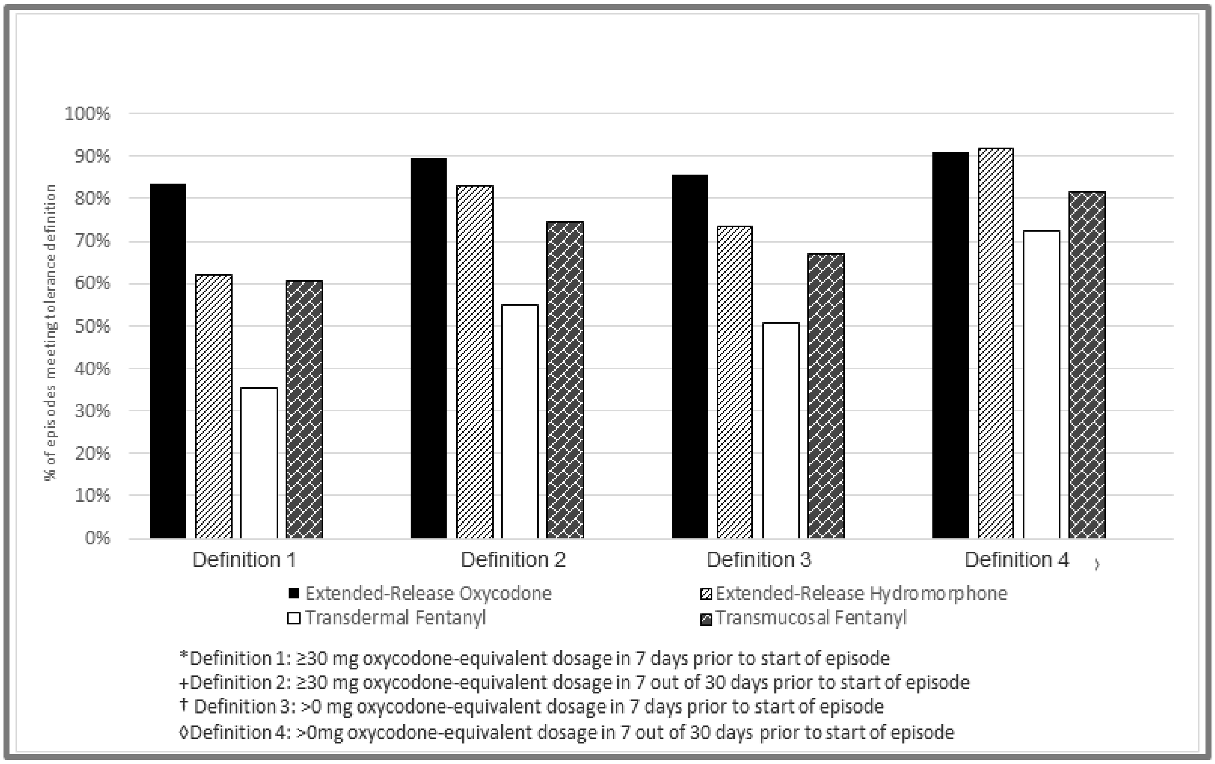

* Note: throughout the following tables and figures, “fentanyl transdermal system” will be shortened to “transdermal fentanyl”

© 2020 Jeffery MM et al. *JAMA Network Open.*

eFigure 2. Proportion of Incident Opioid-Tolerant Only Episodes Meeting Criteria for Tolerance in Medicare Advantage Population

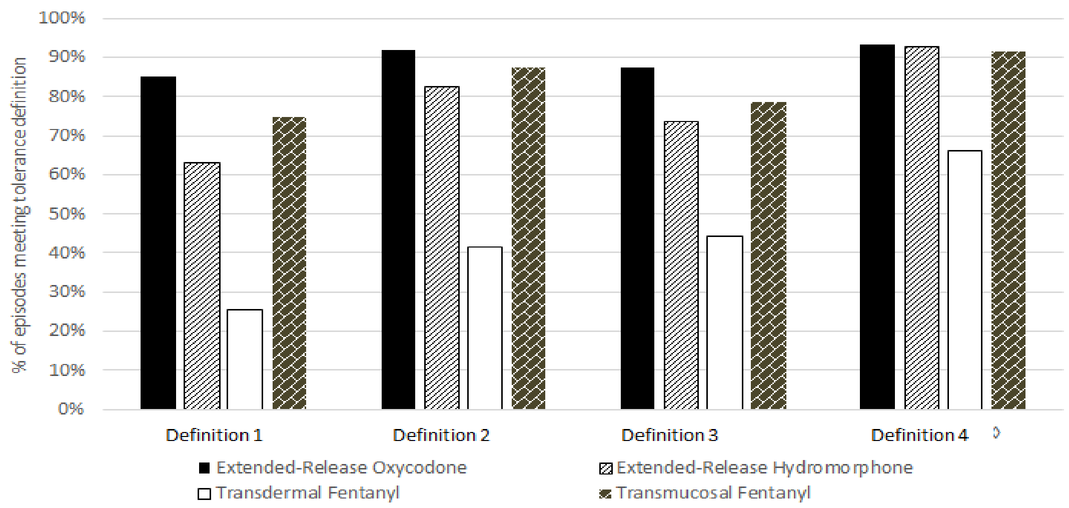

*Definition 1: ≤ 30 mg oxycodone-equivalent dose in 7 day prior
+Definition 2: ≤30 mg oxycodone-equivalent dose in 7 out of prior 30 days
†Definition 3: > 0mg oxycodone-equivalent dose in 7 day prior
◊Definition 4: > 0 mg oxycodone-equivalent dose in 7 out of prior 30 days

Structured EHR data analysis

© 2020 Jeffery MM et al. *JAMA Network Open.*

eTable 5. Opioid Tolerance in Opioid-Tolerant Only Episodes Identified in Electronic Health Record Structured Fields (Main Analysis)

	ER Oxycodone	ER Hydromorphone	Transdermal Fentanyl	Transmucosal Fentanyl	Total
Total Episodes	4,881	835	14,062	266	20,044
Meeting tolerance definition 1	230 (5%)	46 (6%)	341 (2%)	11 (4%)	628 (3%)
Meeting tolerance definition 2	287 (6%)	59 (7%)	509 (4%)	14 (5%)	869 (4%)
Meeting tolerance definition 3	256 (5%)	52 (6%)	511 (4%)	13 (5%)	832 (4%)
Meeting tolerance definition 4	325 (7%)	68 (8%)	740 (5%)	19 (7%)	1152 (6%)

eTable 6. Opioid Tolerance in Opioid-Tolerant Only Episodes Identified in Electronic Health Record Structured Fields

(Secondary Analysis)

	ER Oxycodone	ER Hydromorphone*	Transdermal Fentanyl	Transmucosal Fentanyl*	Total
Total Episodes	113	62	750	14	939
Meeting tolerance definition 1	78 (69%)	>31 (>50%)	191 (25%)	<11	311 (33%)
Meeting tolerance definition 2	90 (80%)	>39 (>63%)	287 (38%)	<11	427 (45%)
Meeting tolerance definition 3	83 (73%)	>36 (>58%)	292 (39%)	<11	422 (45%)
Meeting tolerance definition 4	97 (86%)	45 (73%)	426 (57%)	11 (79%)	579 (62%)

* Some numbers not displayed to mask small cell sizes and comply with cell suppression policies

© 2020 Jeffery MM et al. *JAMA Network Open.*

Additional results: claims analysis

Tolerance by age category

© 2020 Jeffery MM et al. *JAMA Network Open.*

eFigure 3. Opioid Tolerance Claims Analyses Stratified by Age and Opioid-Tolerant Only Medication

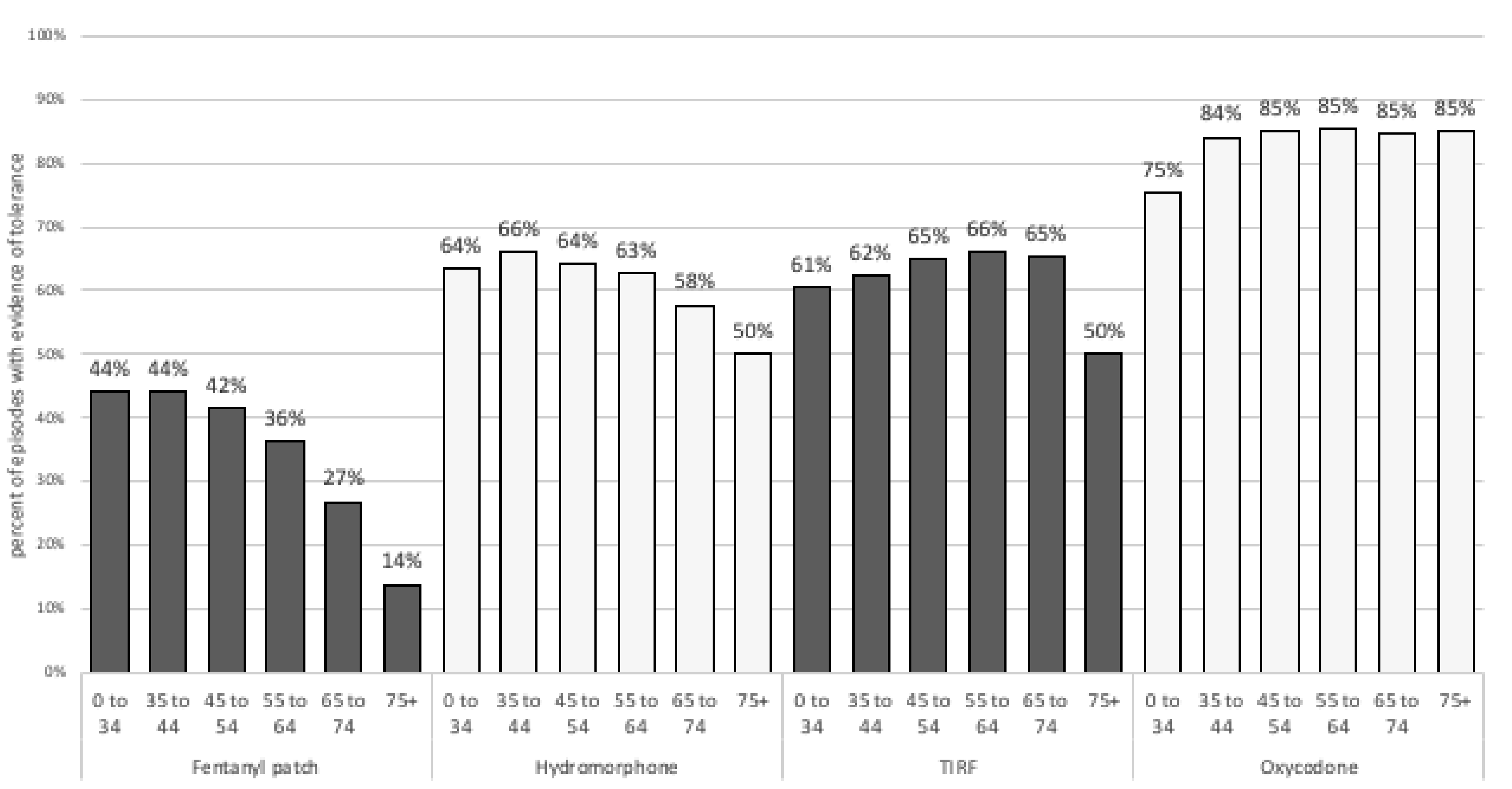

Notes: TIRF=Transmucosal Immediate Release Fentanyl; Oxycodone includes ER Oxycodone at daily dose >80 mg or strength of 60 or 80 mg; uses Tolerance definition 1 (>=30 mg oxycodone equivalents for each of the 7 days before the first opioid tolerant only medication fill)

© 2020 Jeffery MM et al. *JAMA Network Open.*

Opioid tolerance trends

eTable 7. Annual Trends of Evidence for Primary Opioid Tolerance by Opioid-Tolerant Only Type in OptumLabs Data Warehouse

	Extended-Release Oxycodone			Extended-Release Hydromorphone			Transdermal Fentanyl			Transmucosal Fentanyl		
Year	Total (N)	Meeting Primary Tolerance (N, %)		Total (N)	Meeting Primary Tolerance (N, %)		Total (N)	Meeting Primary Tolerance (N, %)		Total (N)	Meeting Primary Tolerance (N, %)	
2007	5,203	4,135	79%	0	0	0%	10,264	3,004	29%	541	318	59%
2008	5,840	4,607	79%	0	0	0%	10,090	2,911	29%	303	152	50%
2009	6,075	4,863	80%	0	0	0%	9,350	2,829	30%	211	118	56%
2010	5,579	4,593	82%	68	52	76%	9,736	2,977	31%	225	135	60%
2011	4,804	4,112	86%	807	508	63%	10,197	3,158	31%	184	117	64%
2012	5,272	4,583	87%	1379	877	64%	10,827	3,367	31%	137	98	72%
2013	3,591	3,196	89%	1711	1030	60%	11,380	3,497	31%	243	190	78%
2014	2,904	2,615	90%	794	504	63%	10,087	3,229	32%	296	206	70%
2015	2,374	2,134	90%	667	404	61%	10,248	3,312	32%	230	173	75%
2016	1,917	1,758	92%	284	193	68%	9,497	3,108	33%	70	54	77%

© 2020 Jeffery MM et al. *JAMA Network Open.*

eFigure 4. Annual Trends in Evidence of Opioid Tolerance in Commercial Episodes in OptumLabs Data Warehouse

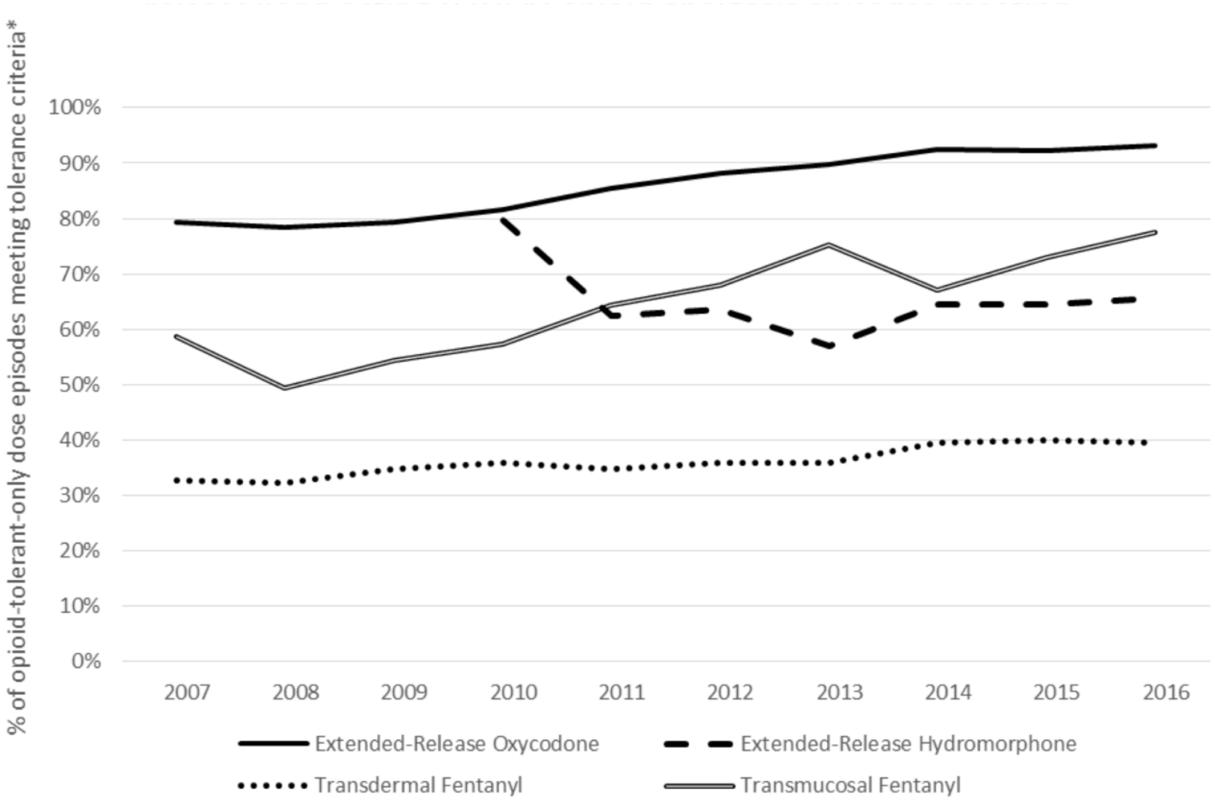

* ≥30 mg oxycodone-equivalent dosage in 7 days prior to start of episode

© 2020 Jeffery MM et al. *JAMA Network Open.*

eFigure 5. Annual Trends in Evidence of Opioid Tolerance in Medicare Advantage Episodes in OptumLabs Data Warehouse

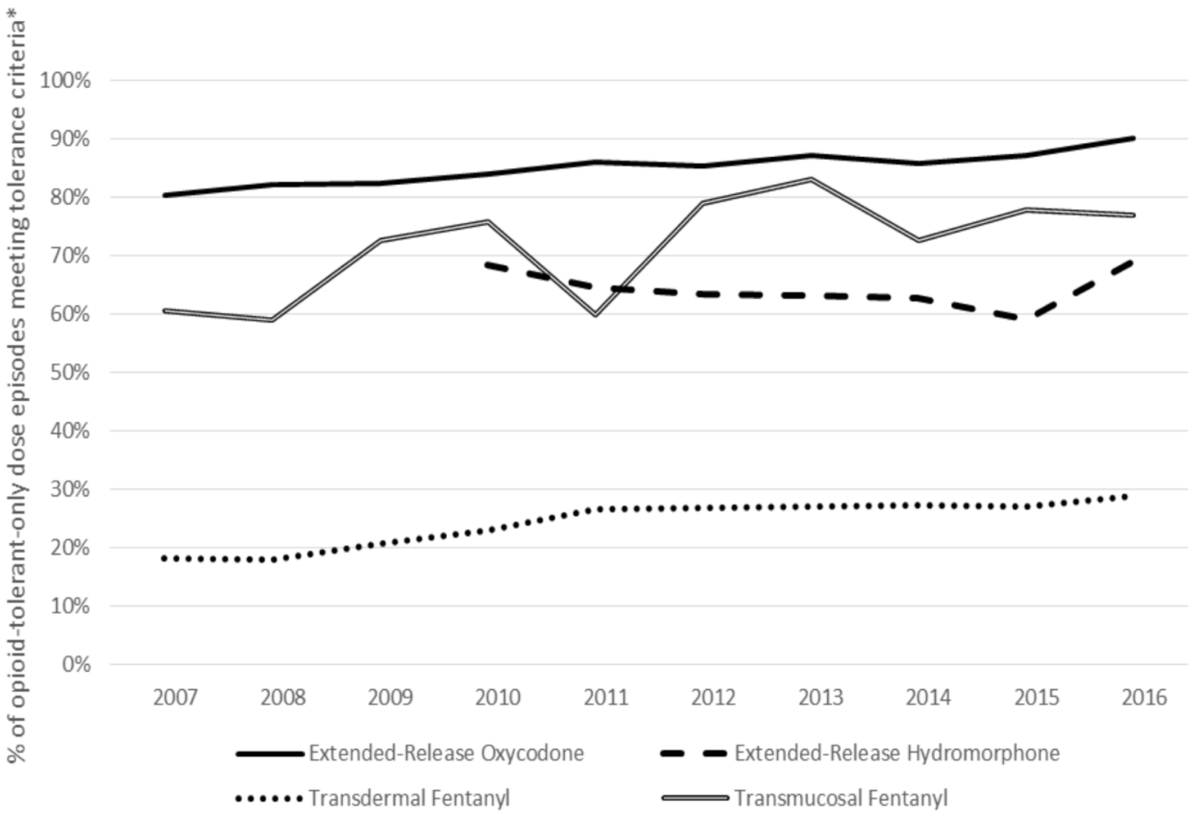

* ≥30 mg oxycodone-equivalent dosage in 7 days prior to start of episode

© 2020 Jeffery MM et al. *JAMA Network Open.*

eFigure 6. Annual Trends in Evidence of Opioid Tolerance in Overall Episodes in OptumLabs Data Warehouse

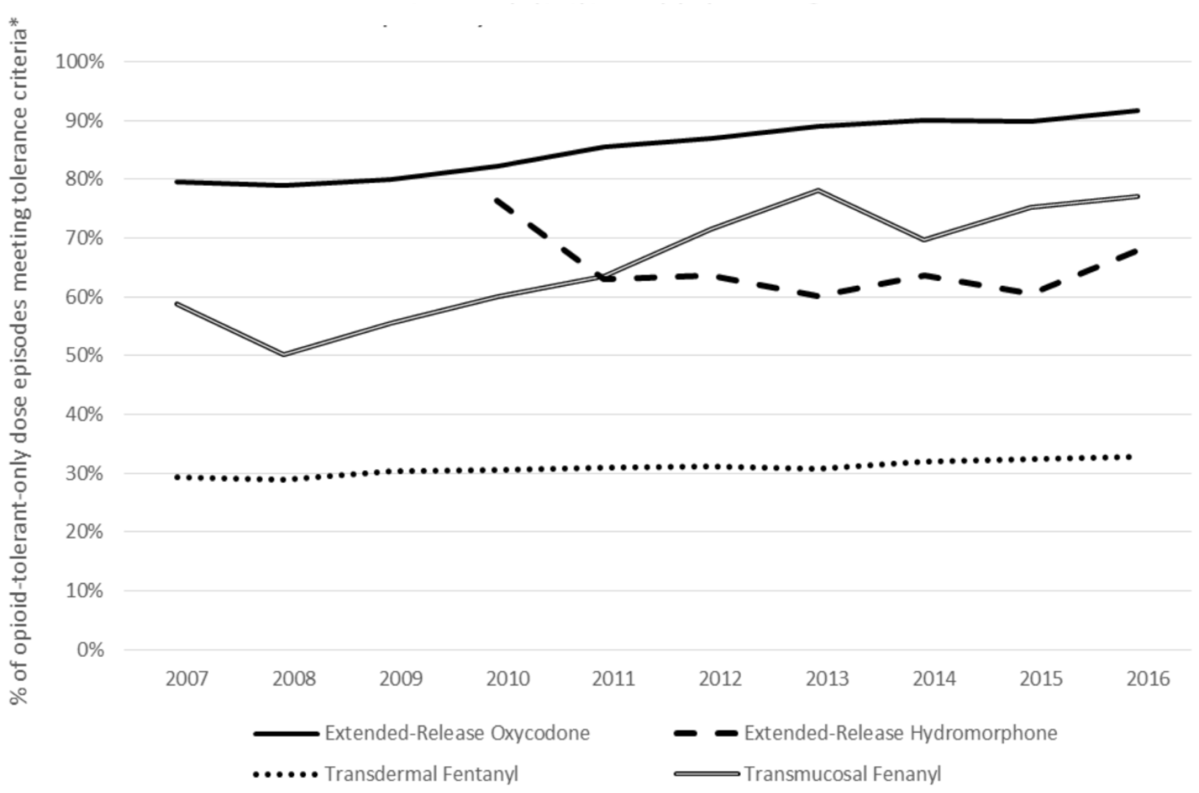

* ≥30 mg oxycodone-equivalent dosage in 7 days prior to start of episode

Natural language processing analysis

Because up to 80% of the information contained in the EHR/ clinical record is stored as free text in clinical notes, the analysis that included free-text EHR notes (henceforth "free-text analysis") utilized natural language processing -- a branch of artificial intelligence that helps computers understand, interpret, and manipulate human language – and applied it to help evaluate the content and meaning of clinical notes. The goal was to determine whether there is additional evidence of opioid tolerance in clinical notes, in addition to that seen in claims or EHR structured fields. Based on feedback received from our technical expert panel, we extended this beyond just looking for evidence of tolerance and also looked for clinical rationale for the

© 2020 Jeffery MM et al. *JAMA Network Open.*

prescribing behavior—that is, potential reasons the clinicians may have prescribed OTO medications to people who were not opioid-tolerant.

The free-text analysis occurred behind an “identifiable” data firewall and had to be conducted without access to claims data. OTO episodes and tolerance were identified according to the data available behind that firewall.

The general approach for cohort definition for the free-text analysis was to create sets of notes highly distinguishable from one another that are sound, though not necessarily complete due to the “leakage” in the EHR described above under the heading Description of Electronic Medical Recorddata

Notes for each OTO event for which notes were available were combined for the 30 days preceding the OTO into a single large text block to use as a record of patient history and encounters.

Definitions

Standard access to the EHR data included in the OLDW does not allow direct access to the clinical notes. The free-text analysis occurred behind a firewall without access to claims and used somewhat different procedures to identify OTO episodes. Specifically:

- Extended-release (ER) and immediate-release (IR) opioids were identified using a list of NDC codes from the CDC(1)
- OTO Episodes were identified using EHR prescriptions written data showing prescribed OTO medication (ER oxycodone; ER hydromorphone; fentanyl transdermal system; or transmucosal fentanyl) with no other extended-release or long-acting opioid exposure in the preceding 183 days

© 2020 Jeffery MM et al. *JAMA Network Open.*

- Opioid *exposure* was used as a proxy for opioid *tolerance* and defined as any opioid exposure in the 30 days preceding an OTO episode
- Opioid exposed group - Those given an ER opioid after a having an IR opioid in the prior 30 days are the exposed group
- Opioid unexposed group – no opioid use of any kind identified in the EHR in the 30 days prior to the OTO episode

Topics: Clusters of terms that:

- frequently occur together or
- frequently occur in similar patterns

Opioid Exposure and Clean Periods

We used a conservative approach for identifying notes for those in the exposed and unexposed groups. Recognizing constraints imposed on the analyses resulting from the diversity of the structured data collection (data is sourced from many health systems and many EHR instances from several EHR vendors), the range of documentation processes of the contributing client organizations, and the potential incompleteness of any patient's clinical history beyond a single encounter, we used the following conservative definitions:

- "Clean" periods were defined as 183 days with no OTO exposure (based on NDC codes), either prescription or administration. This conservative approach of a 183 day time frame was used to be consistent with the claims and claims + EHR structured fields analyses as well as to counteract potential misclassification of the data.
    - Days' supply was often missing for prescription data so we assumed a prescription could be up to 90 days.

© 2020 Jeffery MM et al. *JAMA Network Open.*

- It was not always possible to determine whether refills occurred
- The assumption was that a 183 day clean period without any OTO would filter out any prior tolerance

- We used NDC codes to identify OTO drugs ER hydromorphone, fentanyl transdermal system, transmucosal fentanyl, and ER oxycodone
- Episodes are based only prescriptions written. Medications administered in a hospital setting were not used to identify OTO medication episodes but were used to identify tolerance (exposure).

The first step of the NLP work was to create a meaningful set (or sets) of case (exposed) and control (unexposed) episodes that could be compared. The definitions need to be precise enough to allow a small signal of tolerance to emerge from all the text that was available at the episode level. Using too many notes could have the potential to dilute this signal to the point of non-detection. Because the choice of the case/control definitions was critical to the success of the project, we used a narrow definition as a first experiment, and then expanded in subsequent iterations. Because medication dose and duration cannot be utilized to create tolerance definitions similar to those used in the claims analysis, we defined prior opioid use in this phase as “opioid exposure” rather than opioid tolerance. We extracted the cohort of OTO episodes then divided the cohort into opioid-exposed or “cases” and opioid-unexposed or “control” groups based on whether they had any opioid exposure in the 30 days preceding the OTO episode date.

- OTO episodes were defined as an OTO prescription written, preceded by 183 days free of prior OTO prescriptions or administration
- Case/Exposed group: Those with any IR opioid exposure in the 30 days prior to the OTO date were “cases”

© 2020 Jeffery MM et al. *JAMA Network Open.*

- Unexposed group: Those who were completely opioid naïve in the 183 days prior to the OTO were “controls”

The cohorts were developed using structured EHR data for prescribed and administered drugs.

Free-text Analysis Study Sample

The NLP cohort was created from 10 years of patient records, from 2007-2016, in the notes database. All drug orders, both prescribed and administered, matching the 22,397 opioid NDC codes in the project data dictionary, were collected for IR- and ER oxycodone, transdermal fentanyl, ER hydromorphone, and transmucosal fentanyl prescriptions. The final cohort of OTO episodes consisted of only those prescriptions for OTO opioids for patients with episodes where there were no OTO opioid orders in the database in the prior 6 months.

Natural Language Processing Steps

Clinical notes differ from many types of standard text and require pre-processing to clean the notes (e.g., remove white space and punctuation, identify and remove template language and copy-paste of previous comments), prior to use. Standard pre-processing steps were followed, see Data Cleaning section below for detail.

We used the vector space model, also known as the “bag-of-words” approach, which is a commonly used method of document classification. In this method the frequency with which each word occurs is used as a feature for training for the classifier to identify and categorize the words. A list of words and terms was created, and the number of times each term appeared in each document patient note was counted. See Term Vector Creation section below for examples.

The patient-by-patient counts of how often each term appears in each patient’s notes are scaled by how many other patients also had that term. This scaling makes the data more precise as a

© 2020 Jeffery MM et al. *JAMA Network Open.*

term with a high frequency may be common but not be very specific. The goal was to identify specific words that appear in relatively few documents, but seem important where they do occur (i.e., they occur frequently). The term frequency (TF) is multiplied by its inverse document frequency (IDF) to arrive at the final number used to create the topic. Words or terms were dropped if they occurred in a) less than 1% of notes and were considered rare or b) more than 80% of notes and were considered common.

We used both a combination of machine learning, allowing the computer algorithm to determine the words, and a guided approach where we searched for particular words. The words we specified in the guided approach were based on input from our technical expert panel.

Topic Modeling

For notes analysis we utilized non-negative matrix factorization, a non-parametric method that works well with the bag-of-words approach. The parameters are set to use regularization to reduce topic noise. We used topic modeling, a type of statistical model for discovering the abstract "topics" that occur in a collection of documents that is frequently used in machine learning and NLP. This method is an often used text-mining tool for discovery of hidden semantic structures in a text body. Terms were combined into clusters, called “topics,” and those topics were then weighted and assigned to relevant notes for analysis.

The topics were created by the following steps:

- Identify a cohort of patients with OTO episodes
- Extract 30 days of notes prior to the OTO fill date
- Use NLP algorithm to group the terms found in those notes into “topics” which are clusters of terms or words that either frequently appear together or that occur in similar contexts, seeming to act as synonyms; no human input goes into the creation of topics (i.e., it is unsupervised machine learning)

© 2020 Jeffery MM et al. *JAMA Network Open.*

- Identify which topics are important in each patients' notes (unsupervised)
- Review the topics for meaning with the technical expert panel, describing what the topic represents (e.g., "palliative care" or "surgery")
- Combine the NLP topics with the claims data to determine which topics discriminated between episodes with evidence of tolerance in claims data and those without evidence of tolerance in claims data

The final model specified 100 topics; we also ran models of 50 topics and 200 topics. The 50-topic model resulted in topics that were too general, while the 200-topic model resulted in topics that were too similar to each other. Words that appeared on less than 1% of the notes were dropped, as were topics with an excessive number of terms (top 10th percentile of term counts). Once topics were created they were weighted and assigned back to the relevant notes. After these adjustments, there were 66 topics in the topic model. See **Error! Reference source not found.** for the complete list of 100 topics.

Technical Expert Panel (TEP) Input

After initial exploratory work in NLP we presented preliminary results to our TEP and solicited input. Specific questions (see below) were asked of the TEP as a way to help us understand clinical relevance and clinician documentation habits related to OTO episodes:

- What sorts of complaints, concerns, and scenarios might a physician hear from a patient or caregiver that would lead him/her to consider prescribing an OTO formulation?
- What questions might a physician ask of patients or caregivers that would probe for the OTO being a possible right fit for the individual patient's situation?

© 2020 Jeffery MM et al. *JAMA Network Open.*

- What documentation might exist in progress notes (including labs, clinical indicators, pain level, pain management, medication history) that would help identify the physician's rationale for prescribing?
- What are the specific phrases and terms the notes would have?

In addition to reviewing our topics and providing additional clinical terms that might serve as evidence of tolerance, the experts also suggested we looked for explanatory topics that might provide insight into the physician's rationale for why labelling instructions were not followed. The reasoning might not represent appropriate or safe clinical care. The goal was simply to assess possible explanations for the prescribing.

The TEP suggested a number of reasons for prescribing OTO medication in those without prior opioid tolerance including:

- Patient-related issues
    - Vomiting/nausea
    - Esophageal dysfunction
    - Dementia with inability to follow dosing instructions
    - Attempt to relieve polypharmacy
    - Sleep disruption due to inadequate pain control
- Clinician-related issues
    - Knowledge and training
        - Taught that ER formulations are superior to IR formulations for some patient groups

© 2020 Jeffery MM et al. *JAMA Network Open.*

- Inadequate training about pain management and opioid prescribing
- Lack of knowledge of importance of opioid tolerance when initiating ER opioids and TIRFs; risk evaluation and mitigation strategy (REMS) message not known by everyone in prescribing community [supported by temporal trend toward increased evidence of tolerance]

- Anecdotally, may use ER so the attending physician won't be called to manage pain

This exercise resulted in the creation of a "white list" of additional words and phrases to be utilized. A sample of those words is illustrated below; for the full lists, see **Error! Reference source not found.** through **Error! Reference source not found.**.

Words on this explanatory list were retained regardless of whether they met the 1% threshold required for other clinical terms.

© 2020 Jeffery MM et al. *JAMA Network Open.*

eTable 8. Sample of White List Words Generated by the Technical Expert Panel

Sample "white list" words	
ADL difficulty	Diarrhea
Aide	Difficulty swallowing
Bariatric surgery	Irritable bowel
biliopancreatic duodenal switch	Malabsorption
Breakthrough	Memory
Cancer	Needs help with meds
Cognitive	Palliative
Comfort	Sickle cell
Complex setting	Sleep disruption
Developmental disability	Substance abuse
Difficulty with oral medications	Vomiting

Term Matrix

Finally, a term matrix including both clinical and whitelist terms was created within the secure NLP area and then transferred back into OLDW where it could be merged in with claims and structured EHR data for final analyses. This matrix included patient ID, episode identifier, term, and the number of times the term appeared in the note.

Notes Extraction

Clinical notes in the data set are not available from all providers for all years due to organizational shifts and variation that may occur over time in clinical documentation practices and potentially other factors. The clinical notes for each of the patients for 30 days prior to the OTO episodes were collected. Notes in our dataset are created individually in each provider's EHR, and a single day can produce dozens of notes for a patient while a single episode's 30-

© 2020 Jeffery MM et al. *JAMA Network Open.*

day history can produce hundreds of notes depending on documentation practices and EHR system variation. For inpatient stays, one note may be just an update of vital signs. We chose to merge all notes for a single episode into one document that could be used to evaluate the text that created topics, i.e. we processed text at the episode level. By aggregating the notes to their episodes there is a consistent unit of analysis, and we avoid the complexity of trying to attribute meaning to an episode from a multitude of notes.

Data Cleaning

NLP requires some basic cleaning and processing to make the notes more useful. Punctuation, white-space, proper names, and very common words (e.g., "the" "and") are removed. These things do not provide useful information, for example it is not possible to distinguish context for very common words.

Many notes in an EHR record are cut-and-pasted from prior notes for the same patient, resulting in a complete history of all prior notes with minimal new information added to each new note created. This can cause over counting of terms in the original note, as they would be repeated many times. An example of how cut-and-paste might look in a note is below:

eTable 9. Example of Copy and Paste

Note 1	*Patient has a complaint of fever.*
Note 2	*Patient has a complaint of fever.*<br>*Allergies:*<br>*Temp=100.3*
Note 3	*Patient has a complaint of fever.*<br>*Allergies: None*<br>*Temp=100.3*

Only the last note would be kept as it contains complete information. This filtering process reduced our total note counts by 11% but the number of episodes and patients remains the same

Often notes also include language from standard templates. These include standard surveys, discharge instructions, and phrases repeated for all patients at that provider, with no information specific to any individual patient. These are often form data logged into the note as well as answers. Hospitals and providers have their own standard templates that may be included for all patients seen at that facility. Frequently seen examples of this in our data included:

- Diet/Nutrition: Regular Diet: Eat a wide variety of foods including fresh fruits and vegetables, whole grains, lean meats, poultry and fish and low-fat dairy products.
- Pain: Medications - see medication list. Measures to relieve pain reviewed. Call your doctor for any new onset of pain, change in intensity or quality of pain or change in the ability to do your activity.
- Review of Systems:
    - Denies fever, chills, or sweats
    - Denies blurred vision, diplopia, change in visual acuity
    - Denies hearing loss, nasal congestion, sore throat
    - Denies chest pain, syncope, or palpitations
    - Denies dyspnea, cough, PND

Removing common templated phrases reduced noise and improved our ability to extract content to build meaningful topics from the notes. Otherwise, specific terms may be far removed from other words signaling their negation and processed inappropriately. Not removing them may cause the above patient to be included in a cluster with topics of "*blurred vision*," "*hearing loss*" and "*chest pain,"* though the patient has none of those symptoms. Because of facility-specific templates, early processing of the notes without this step found notes clustered by hospital group, not by patient specific content. We looked for common phrases with a minimum of 7 words and redacted those phrases. This processing does not change the number of episodes, it

only reduces the volume of text per episode, but leads to having more meaningful text to evaluate.

Term Vectors and Clusters

The next stage of processing was to create term vectors from the data set. A term vector is a count of phrases in a text document.

Creating vectors of single word phrases for the sentence:

NOTE: He is receiving fentanyl since he has been here for back pain, leg pain, abdominal pain, and pain in the feet.
Common words such as *he*, *is*, *and*, and *the* are ignored and the note converts to:

eTable 10. Single-Word Phrase Vector

abdominal	feet	fentanyl	leg	pain	receiving
1	1	1	1	4	1

and a vector of 1-2 word phrases would be

eTable 11. Multiword Phrase Vector

abdominal	abdominal pain	feet	fentanyl	fentanyl pain	leg	leg pain	pain	pain abdominal	pain feet	pain leg	receiving	receiving fentanyl
1	1	1	1	1	1	1	4	1	1	1	1	1

Creating a Matrix from Vectors

Vectors for several sentences or documents generate a matrix, and the rows of the matrix can be compared for similarity and grouped into clusters. The matrix excludes common or rare phrases. For example, these phrases in **Error! Reference source not found.** can be converted into the vectors in **Error! Reference source not found.** below.

© 2020 Jeffery MM et al. *JAMA Network Open.*

eTable 12. Phrases to be Converted Into Vector Matrix

She is on oxycodone and has difficulty with sleep due to back pain.
She feels that her activities of daily living are increased, but she is still on the oxycodone and methadone.
His other medicines include oxycodone, an aspirin a day, Prilosec, Dilantin and Flagyl.
Fentanyl gives him at least three hours pain relief. He has significant problems with fractured sleep.
Current medications include enalapril, low dose enoxaparin, Fentanyl patches. He is no longer on fluconazole.
He has chronic back pain and a fentanyl patch. He denies any constipation, diarrhea, abdominal pain.
He is receiving fentanyl Since he has been here for back pain, leg pain, abdominal pain, and pain in the feet.
He states that he is currently in pain and the fentanyl only helps for about an hour or so before the pain resumes.
Dr. Smith has maintained him on opioid medications consisting of Norco 10/325 mg for breakthrough pain and oxycodone.
The patient has not tolerated morphine in the past. We will start oxycodone 5 mg q.2h. as needed.

eTable 13. Vector Matrix of Phrases in eTable 12

abdominal	fentanyl	include	medications	mg	oxycodone	pain	sleep
0	0	0	0	0	1	1	1
0	0	0	0	0	1	0	0
0	0	1	0	0	1	0	0
0	1	0	0	0	0	1	1
0	1	1	1	0	0	0	0
1	1	0	0	0	0	2	0
1	1	0	0	0	0	4	0
0	1	0	0	0	0	2	0
0	0	0	1	1	1	1	0
0	0	0	0	1	1	0	0

When describing groups of episodes, phrases that are too common (e.g., *patient)* or too rare (e.g., *Canavan Disease*) are ignored by setting minimum and maximum frequency parameters. For this project those filters were set to 80% and 1%, respectively.

© 2020 Jeffery MM et al. *JAMA Network Open.*

At this point, the episode data is contained in a term-frequency matrix. Each row of this matrix is an episode, each column is a term and the values in the matrix are the frequency counts. These counts are then multiplied by the inverse document frequency (how many episodes contain the term) to adjust the matrix so terms that are specific to only a few documents (i.e., notes) have more relevance. (Salton & Buckley, 1988).

Clustering patients or other data is usually an assignment of the observation to a single cluster that is performed to maximize similarity within each cluster while maximizing separation of the clusters. With NLP data, and term-frequency, inverse document frequency (TF-IDF) matrices, clustering is more often a weighted assignment of patients to many clusters. In this case, a patient has a weight that indicates the importance of that cluster in the patient's data. The methods for this type of clustering come from analyzing text documents where the clusters are called topics, each episode's TF-IDF vector is a weighted collection of topics like back pain, prescription orders, physical therapy etc.

The approach chosen for topic creation is non-negative matrix factorization, where the TF-IDF matrix is factored into two matrices, an episode-topic and topic-term matrix. Because the TF-IDF is non-negative, each matrix element can be approximated as a non-negative linear combination of topic weights and term weights.

Given the original matrix **A**, we can obtain two matrices **W** and **H**, such that **A**= **WH**. Non-negative matrix factorization has an inherent clustering property, such that **W** and **H** represent the following information about **A**:

A (Document-word matrix)—input that contains which words appear in which documents.

W (Basis vectors)—the topics (clusters) discovered from the documents.

H (Coefficient matrix)—the membership weights for the topics in each document.

© 2020 Jeffery MM et al. *JAMA Network Open.*

The number of topics is pre-selected. The project computed 25, 50 and 100 topics. After review by experts, 100 topics were chosen as sufficiently many to have precise topics (e.g., sickle cell disease, diabetes, lung cancer, and knee surgeries appear in separate topics without other conditions) without having duplicative topics (separate topics for 'oral' vs 'orally').

Free text analysis Results

Starting with a corpus of almost a billion notes from 2007-2016, we identified opioid episodes by opioid type and then applied inclusion/exclusion criteria. This left us with a cohort of 168,916 OTO episodes which occurred in 149,408 individuals. This cohort was further broken into two groups: 99,761 episodes contributed by 89,163 individuals had no record of an opioid prescribed or administered in the preceding 30 days, which were labeled as unexposed, or "controls"; 69,155 episodes contributed by 65,526 individuals had evidence of an IR opioid in the preceding 30 days and were labeled as exposed, or "cases."

eTable 14. Clinical Notes Cohort

Cohort Description	Prescriptions	People
All opioid orders 2007-2016	43,290,736	6,334,017
All OTO prescriptions written	1,462,520	187,489
OTO prescriptions with 183 days clean of OTO	168,916	149,408
Unexposed to IR opioid in 30 days	99,761	89,163*
Exposed to IR opioid in 30 days	69,155	65,526*

*Individuals may have >1 prescription so the people column will not sum

The notes cohort was similar to the claims and EHR structured fields cohorts on gender and age (see **Error! Reference source not found.**).

© 2020 Jeffery MM et al. *JAMA Network Open.*

eTable 15. Demographics of Clinical Notes Cohort

Cohort Description	Episodes	Episode %	Individuals*	Individuals %
OTO Episode	46,525	100.0%	42,868	100.0%
Male	19,745	42.4%	18,341	42.8%
Female	26,780	57.6%	24,527	57.2%
Age*				
0-17	228	0.5%	222	0.5%
18-24	775	1.7%	748	1.7%
25-34	2,857	6.1%	2,683	6.3%
35-44	4,945	10.6%	4,598	10.7%
45-54	9,116	19.6%	8,489	19.8%
55-64	10,916	23.5%	10,143	23.7%
65-74	8,539	18.4%	7,981	18.6%
≥75	9,149	19.7%	8,592	20.0%

*Individuals may have >1 episode so age groups will not total the number of individuals

We created a matrix of words and the frequency with which each appeared in each episode (see **Error! Reference source not found.**). The matrix data included 46,525 episodes and over 12,000 terms. We evaluated which topics were associated with opioid exposure in the 30 days prior to the OTO episode. The most commonly occurring included topics related to vital signs, medication instructions, and cancer. The least commonly occurring included topics related to medications and imaging.

© 2020 Jeffery MM et al. *JAMA Network Open.*

eTable 16. Example of Topics Before Cleaning Generated From Clinical Notes

Topic #	Label	Top 5 - Topics most associated with opioid exposure
Topic #52	Vital signs	maximum temperature, temperature, maximum, temperature temperature, temperature maximum, exception maximum, maximum maximum, respiratory, exception, oxygen
Topic #45	Medication instructions (discharge)	instructions, every instructions, two, daily, daily instructions, every, two instructions, three, four, free entry
Topic #88	Metastatic cancer	metastatic, lung, chemotherapy, radiation, cancer, mass, adenocarcinoma, lobe, lung cancer, scan
Topic #69	Medication order	filled, oral filled, ph fax, ph, eligible details, member eligible, systems, fax, onset, none
Topic #51	Squamous cell carcinoma	squamous cell, cell carcinoma, squamous, carcinoma, cell, radiation, radiation therapy, tongue, chemotherapy, cisplatin
**Topic #**	**Label**	**Bottom 5- Topics least associated with opioid exposure**
Topic #13	Oral meds, extended release	orally, oral orally, oral, orally every, release orally, discharge, release, extended release, reconciliation, extended
Topic #72	Inpatient IV	oral oral, intravenous, mcv hct, hb mcv, piggyback, gap wbc, anion gap, anion, gap, mcv
Topic #4	Medication review	rpt, daily rpt, oral rpt, every rpt, signature electronically, ebody, rec list, signature, list reconciled, rpt oral
Topic #30	Fentanyl patch	patch, fentanyl, patch every, apply patch, apply, patch apply, transdermal, transdermal patch, fentanyl patch, every
Topic #86	Diagnostic imaging	mrn yrs, accession order, yrs sex, interpreted approved, sex adm, electronically diagnostic, imaging complete, diagnostic imaging, interpreted, accession

The topics and matrices were brought back into the OLDW environment and linked with the claims and structured EHR data. Because the EHR data only partially overlap with claims, and not all facilities that contribute EHR data include patient notes, we did not have full notes/NLP data on all claims-defined episodes (**Error! Reference source not found.**).

eTable 17. Final Topics Matrix Cohort After Merging With Claims

Claims episodes	153,385	% of prior line
Unique ID in episodes	131,762	
Also appear in the clinical patient table at any time	40,121	30.40%
And notes eligible (facility submits notes with EHR data)	37,651	93.80%
And has a note at any time	26,535	70.50%
And has an opioid prescription written at any time	21,524	81.10%
And has an opioid prescription within +/- 90 days of claims episode	8,534	39.60%
And has ≥1 OTO specific prescription note in the 30 days prior to the OTO prescription)	514	6.00%
Episodes where the note prescription date is within +/- 7 days of the claims Rx date	249	48.40%

Because our final sample size was relatively small, we collapsed terms into themes to minimize null cells in our analysis. The distribution of terms and whitelist words was not different between the populations that were tolerant or non-tolerant. We calculated risk ratios for the term categories as the risk of non-tolerance for episodes that included the term divided by the risk of non-tolerance for episodes that did not include the term. A risk ratio (RR) greater than 1 indicates that people with the term were more likely to be non-tolerant. None of the RRs for term categories were statistically significant. *Patient request* had the largest RR (1.24 [95%CI 0.92 to 1.67]), and *pain severity* had the lowest RR (0.89 [95% CI 0.75 to 1.06]).

© 2020 Jeffery MM et al. *JAMA Network Open.*

eTable 18. Risk of Nontolerance Among Patients With and Without Terms, With Both Claims and Free-Text Notes Electronic Medical Record Data

Term	Risk Ratio	Lower Confidence Interval	Upper Confidence Interval	Count in non-Tolerant	Count in Tolerant
Patient request/affective components	1.24	0.92	1.67	160	57
Vomiting/GI	1.15	0.94	1.41	142	60
Sleep	1.02	0.87	1.19	137	47
Cognitive/functional deficit	0.95	0.81	1.12	116	48
Addiction	0.94	0.80	1.09	92	40
Pain severity/lack of response/can't take other drugs	0.89	0.75	1.06	82	31

Tolerance: Evidence of ≥30mg of oxycodone equivalents on each day of the 7 days prior to OTO episode using claims data, exclusive of start date)

NLP Analysis Summary

Although we started with a large number of OTO episodes based on claims data and a large number of clinical notes, the overlap of notes preceding specific OTO episodes was relatively small. We used NLP to extract information from clinical notes in an attempt to identify evidence of previous opioid tolerance that may not be available in claims data alone. Using topic modeling we evaluated numerous topic cut-points and configurations and were unable to find topics that were indicative of opioid tolerance. Based on feedback from our TEP, we went a step further to determine if we could identify explanatory reasons why clinicians may prescribe in a manner inconsistent with product labeling. We were unable to identify any statistically significant explanatory topics.

© 2020 Jeffery MM et al. *JAMA Network Open.*

Note on protecting patient privacy:

Free-text notes were kept in a separate secure network with unique credentials including two-factor authentication achievable only from a laptop not connected to the de-identified claims and structured EHR data. When the analysis of the clinical notes was completed, the generated data were moved by a separate compliance team from the environment where they were created to another environment with access to claims and structured EHR data. OptumLabs compliance experts worked with the team to ensure no protected health information was transferred from the NLP environment to the de-identified claims/structured EHR data environment.

eTable 19. List of 100 Topics from NMF Model

Topic Name	# Terms with Weights	First 10 Words
**Oral**	6	oral, oral rpt, hcl oral, sodium oral, oral oral, release
**Oral medication**	754	oral, daily, oral daily, hcl oral, release, hcl, release daily, oral extended, extended release, extended
**Communication documentation**	170	sms, preliminary, dictating, billing, clyde, regional clyde, telephone fax, job, regional, soarian
**Medication review**	143	rpt, daily rpt, oral rpt, every rpt, signature electronically, ebody, rec list, signature, list reconciled, rpt oral
**Dispensing Meds/labs**	533	dispersed, order, observation receipt, order number, receipt reported, reported referring, order status, filler order, filler, receipt
**History – med review**	817	history changes, changes required, list, minute, hcl, list includes, critical, required, changes, impression recommendations
**Meaningful use (MU) documentation**	204	satisfied, satisfied record, list satisfied, screening, mu, aco, mu medicare, pqrs, tn, general population
**Brief exam**	398	brief, brief formend, brief formstart, formend, formstart, vstart, vend, pulmonary brief, brief vstart, cardiac brief
**Medication refill**	29	quantity refills, quantity, refills, active quantity, refills none, status active, none, active, orally status, pharmacy

© 2020 Jeffery MM et al. *JAMA Network Open.*

Topic Name	# Terms with Weights	First 10 Words
**Medication dosing/refill**	245	quantity refills, status resolved, quantity, refills, daily quantity, dates details, resolved, dates, started, status
**Back pain**	359	lumbar, spine, back, disc, stenosis, mri, lumbar spine, low back, spinal, fusion
**Oral medication, extended release**	80	orally, oral orally, oral, orally every, release orally, discharge, release, extended release, reconciliation, extended
**Routine Labs-CBC**	156	range, range negative, range range, auto, absolute, range creatinine, wbc range, range mch, range rdw, fl range
**Lab test ranges**	7	low range, high range, low, high, absolute, auto, plasma
**Rehab Assessment**	1,250	therapist, topic, functional, therapeutic, supine, rolling, help another, occupational, another person, much help
**Physical exam**	792	degno, degnormal, degwell, degnot, abnormalities, neurological, degoriented, degoriented place, palpation, cardiovascular
**Telephone refill request**	197	call, phone call, phone, call details, summary call, details, caller, call back, called, call call
**Skeletal fracture**	191	fracture, fractures, distal, orif, femur, fixation, tibia, comminuted, injury, splint
**Oral drug**	6	active orally, orally, oral active, active, release active, cause drowsiness
**Signatures (electronic or handwritten)**	335	electronic signature, handwritten, order, void invalid, signature void, signature must, sheet placed, handwritten order, invalid, handwritten electronic
**Clinic visit summary**	264	clinical summary, phone mrn, mrn address, details clinical, clinical, summary, address, appointment phone, birth, calculated
**Inpatient discharge summary**	1,307	discharge, electronic signatures, instructions, electronic, call, signatures, destination, disposition destination, handouts, discharge disposition
**Anesthesia note**	522	anesthesia, incorrect, epidural, operation incorrect, regional anesthesia, block, perioperative, tissue infection, operation, regional
**Status note**	41	status active, active, daily status, status, formend, formstart, oral status, brief formend, brief formstart, every status
**Lab testing documentation**	65	producer, lab producer, lab, wi, order, expected, observation, acct, neg, campus
**PT or rehab assessment**	548	impairments, details, contributing, resident, disability, weakness decreased, decreased rom, rom, extremities, decreased

© 2020 Jeffery MM et al. *JAMA Network Open.*

**Topic Name**	**# Terms with Weights**	**First 10 Words**
**Medication request and order**	171	oral every, every, oral, generated, every oral, phone fax, phone, type, fax, hcl oral
**Fentanyl patch**	305	patch, fentanyl, patch every, apply patch, apply, patch apply, transdermal, transdermal patch, fentanyl patch, every
**Hospital instructions**	666	reinforced, noninvasive, oi, topic, desc, shift, indicators, environment, fall prevention, intbl
**Special prescription authorization**	145	rxid, authorized, entered authorized, entered, give rxid, give, handwritten rxid, pharmacy, handwritten, daily entered
**Inpatient assessment (falls)**	197	visual, nibp, braden, fall, reassessment, row, respiratory, respiratory respiratory, sedation, temperature
**Prescription/refill**	931	every oral, oral entered, entered, oral, ph fax, every, ph, refills pharmacy, fax, pharmacy
**Inhaler medications**	103	inhalation, inhale, inhalation aerosol, aerosol, puffs, inhale puffs, aerosol inhale, hfa, hfa base, base inhalation
**incision**	1	incision
**Lab tests**	196	auto, rel, shared laboratory, rmg shared, rmg, auto rel, shared, laboratory filler, hisscp, hisscp lab
**Medication review/reconciliation**	32	active standard, standard, routine active, routine, active, oral routine, discontinued standard, standard oxycodone, constipation active, daily routine
**Right knee issue**	5	right, right knee, right lower, extremity, right upper
**Post anesthesia**	1,994	pacu, measurable, anesthesia, consciousness, prior, directive, kg, electronic signatures, correct, operative
**Breast cancer**	30	breast, breast cancer, cancer, left breast, right breast, mastectomy, metastatic breast, history breast, mammogram, ductal
**Knee surgery**	56	knee, right knee, left knee, arthroplasty, knee replacement, knee arthroplasty, medial, replacement, osteoarthritis, flexion
**Medication instructions (discharge)**	452	instructions, every instructions, two, daily, daily instructions, every, two instructions, three, four, free entry
**ED visit**	686	nibp, triage, instructions, wnl, reportable, disposition, electronic signatures, electronic, c, attending
**External attachment**	29	external, external attachment, attachment type, attachment, image external, type image, imported, external external, image, type

© 2020 Jeffery MM et al. *JAMA Network Open.*

Topic Name	# Terms with Weights	First 10 Words
**Negative uranalysis**	4	negative, negative negative, ua, range negative
**Nursing note**	5	lpn, lpn authorized, pharmacy, lpn phone, lpn wrote
**Squamous cell carcinoma**	95	squamous cell, cell carcinoma, squamous, carcinoma, cell, radiation, radiation therapy, tongue, chemotherapy, cisplatin
**Vital signs**	358	maximum temperature, temperature, maximum, temperature temperature, temperature maximum, exception maximum, maximum maximum, respiratory, exception, oxygen
**denies**	1	denies
**Left extremity**	6	left, left knee, left lower, extremity, left foot, left upper
**Diabetes**	95	diabetes, mellitus, diabetes mellitus, insulin, type diabetes, lantus, type, diabetic, metformin, subcutaneous
**Miscellaneous auto-filled chart text**	531	included findings, historian, key, resp null, null, hg hg, hg, included, null hg, status done
**Assessment opioid use**	418	assessed, assessed unchanged, give rxid, unchanged, level, coordinating, effort, entered authorized, managable level, level intractable
**Urine testing**	20	u, range negative, urology, ua, pvr, degwell, ketone, urinary, clarity, flank
**Multiple myeloma**	55	myeloma, multiple myeloma, marrow, multiple, bone marrow, bone, dexamethasone, igg, lytic, plasma
**Telephone refill request/assessment**	68	wrote, converted flag, converted, flag, lpn wrote, called, wrote called, thanks, please, wrote please
**Automated chart entry - Illinois**	181	il, springfield, il address, dates details, springfield il, details, dates, work phone, hg, address
**Electronic prescription**	15	terms: dispense, daw, n, sufficient, record, transmission, surescripts, system, charles, illness hpi
**Hospice/palliative care**	8	hospice, palliative, terminal, morphine, comfort, concentrate, prognosis, faxed
**Patient controlled analgesia**	74	pca, hydromorphone, adult pca, pca hydromorphone, adult, push, demand, ondansetron, retrievedloxone hcl
**Medication order**	23	filled, oral filled, ph fax, ph, eligible details, member eligible, systems, fax, onset, none
**Lab testing (specific clinic**	203	burlington, serum, labcorp, william, bn, labcorp burlington, burlington nc, court burlington, william hancock, bn labcorp
**Prostate cancer**	16	prostate, prostate cancer, psa, cancer, metastatic, bone, bone scan, radiation, history prostate, radiation therapy
**Inpatient IV**	46	oral oral, intravenous, mcv hct, hb mcv, piggyback, gap wbc, anion gap, anion, gap, mcv

© 2020 Jeffery MM et al. *JAMA Network Open.*

Topic Name	# Terms with Weights	First 10 Words
**Lower extremity**	35	ankle, foot, left ankle, right ankle, ankle fracture, left foot, right foot, malleolus, medial, splint
**Medication prescription/instructions**	16	daily therapy, therapy, every therapy, therapy oral, signatures electronically, therapy allergies, therapy status, therapy omeprazole, therapy aspirin, therapy levothyroxine
**Lab tests, results pending**	24	pending, p, producer, scc, nh, new, memorial, rd, preliminary observation, status preliminary
**Orthopedic – shoulder, upper extremity**	50	rotator, rotator cuff, cuff, tear, cuff tear, biceps, arthroscopic, repair, cuff repair, arthroscopy
**Oxycodone**	22	oxycodone hcl, oxycodone, hcl, oxycontin, oxycontin oxycodone, hcl oxycodone, hcl one, hcl every, hcl oral, every
**Lab – CBC**	136	absolute, expected, gfr, monocytes, neutrophils, basophils, eosinophils, lymphocytes, gfr calculated, absolute eos
**Medication mode**	12	oral therapy, therapy, release therapy, patch therapy, hcl oral, caps therapy, hcl, therapy allergies, therapy oral, signatures electronically
**Vaccine**	78	vis, exp vis, given vaccinator, vaccinator, vis vis, lot mfr, vis given, mfr, exp, amt
**Rheumatoid arthritis**	19	rheumatoid, rheumatoid arthritis, arthritis, methotrexate, prednisone, joints, synovitis, wrists, rheumatology, folic acid
**Specific names**	66	hansen, thomas hansen, ebody, hansen ebody, thomas, duragesic, rebecca, triage, hydrocodone, caller
**Medications list**	12	active, active none, orally active, none, daily active, every active, orally, active history, oral active, kg
**Atrial fibrillation**	35	fibrillation, atrial fibrillation, atrial, coumadin, warfarin, paroxysmal atrial, paroxysmal, pacemaker, anticoagulation, ventricular
**Coronary artery disease**	105	artery, coronary, coronary artery, carotid, aortic, stenosis, bypass, stent, valve, graft
**Diagnostic imaging**	438	mrn yrs, accession order, yrs sex, interpreted approved, sex adm, electronically diagnostic, imaging complete, diagnostic imaging, interpreted, accession
**Chart update – new drugs**	15	added new, added, new, observation, clinical changes, changes added, daily added, clinical, authorized, caps
**Metastatic cancer**	598	metastatic, lung, chemotherapy, radiation, cancer, mass, adenocarcinoma, lobe, lung cancer, scan
**Smoking cessation counseling**	78	overdue, maintenance, every, tobacco status, td, mgmt, daily, secondhand, tobacco, lipid profile

© 2020 Jeffery MM et al. *JAMA Network Open.*

Topic Name	# Terms with Weights	First 10 Words
**Auto chart – non specific**	8	mps, cst, examination, side risks, cst electronically, instructions agrees, possible side, therapy active
**Medication dosing interval**	37	one daily, one, daily, hcl one, hcl, oral one, one every, caps, every, one three
**Auto chart update, flow sheets**	2,762	retrieved, electronic signatures, electronic, oral, signatures, meds retrieved, hb, ca, hct plt, wbc hb
**Tracheostomy/feeding tube**	27	peg, trach, per, feeds, liq, oral liq, tracheostomy, oral per, respiratory, tf
**Auto chart update - labs**	17	range flag, flag, flag h, n, h, serum, bodymessage, signature electronically, ebody, eos
**Routine vitals**	267	hg, noninvasive, vte, rating, braden, oximetry, defined, peripheral, braden braden, systolic hg
**Pulmonary/cardiovascular assessment**	892	assessed, denies, assessed unchanged, unchanged, intercostal retractions, intercostal, retractions, auscultation rales, systems general, muscle intercostal
**Pancreatic conditions**	60	pancreatic, pancreatitis, pancreatic cancer, pancreas, duct, abdominal, pancreatic mass, ercp, biliary, stent
**Vital signs**	1,028	calculated, oral, bmi calculated, calculated bsa, bsa calculated, signatures electronically, height ft, therapy, respiration, ft
**Auto chart update – patient request handling**	134	assigned, edited, previously assigned, bodymessage, electronically, ebody, signature electronically, regarding, reassigned previously, reassigned
**Physical exam**	3,966	chronic, back, daily, would, per, status, chest, skin, lower, weight

eTable 20. Terms in Addiction Category

alcohol drug	denied alcohol	narcotics
alcohol caffeine	drink alcohol	overdose
alcohol consumption	drinks alcohol	personal alcohol
alcohol denied	drug alcohol	quit alcohol
alcohol history	Illegal	smoking alcohol
alcohol illicit	Illicit	alcohol
alcohol intake	illicit drug	
alcohol nondrinker	illicit drugs	
alcohol none	Legal	
alcoholic	Naloxone	
alcoholic beverages	naloxone hcl	
alcoholism	Narcotic	

© 2020 Jeffery MM et al. *JAMA Network Open.*

eTable 21. Terms in Sleep Category

able sleep	sleepiness
ambien	sleeping
asleep	sleeping comfortably
difficulty sleeping	sleeps
falling asleep	sleepy
help sleep	staying asleep
obstructive sleep	trouble sleeping
poor sleep	unable sleep
sleep	wakes
sleep apnea	zolpidem
sleep disorder	zolpidem oral
sleep disturbance	zolpidem tartrate
sleep disturbances	

eTable 22. Terms in Cognitive or Functional Deficit Category

address deficits	Dementia	fall factors	hearing impaired	patient/caregiver
advanced difficulty	Difficult	fall	history falls	s/p fall
aphasia	difficult assess	fall injury	impaired	sensory deficits
arousable	Difficulties	fall prevention	impaired impaired	toilet
attendant	Difficulty	fallen	impairments	toileting
caregiver	difficulty ambulating	falling	increase independence	unstable
caregivers	difficulty breathing	falls	independence	unsteady
cerebrovascular accident	difficulty getting	family/caregiver	independently	
coma	difficulty urinating	focal deficits	loss consciousness	
confused	difficulty walking	frequent falls	mechanical fall	
confusion	Disability	functional independence	motor deficits	
deficit	Disabled	functional status	neurologic deficits	
deficits	Fall	glaucoma	neurological deficits	

© 2020 Jeffery MM et al. *JAMA Network Open.*

eTable 23. Terms in Patient Request or Affective Components Category

active anxiety	anxiety depression	bupropion	dangerous	history anxiety	requesting	uncertain behavior
acute distress	anxiety history	challenging	depressed	history depression	resp distress	upset
aggressive	anxious	chronic depression	depression	history psychiatric	respiratory depression	wanted
aggressively	apparent distress	citalopram	desired	homicidal	seroquel	wanting
agitated	ativan	citalopram hydrobromi de	disorder anxiety	ideation	sertraline	wants
agitation	ativan anxiety	clonazepam	distress	klonopin	shopping	wellbutrin
agreeable	ativan lorazepam	concerned	distressed	lorazepam	suicidal	would prefer
alert cooperative	attorney	cooperative	duloxetine	personal behavioral	suicidal homicidal	xanax
alprazolam	behavior	Coping	emotional	psych depression	supportive counseling	
amenable	behavioral	counseling	fear	psychiatric anxiety	tearful	
anger	believes	cpep duloxetine	feeling depressed	psychotic	toradol	
anxiety	bipolar	Crisis	fluoxetine	quetiapine	trazodone hcl	
anxiety chronic	bipolar disorder	daily lorazepam	generalized anxiety	refused	unable obtain	

© 2020 Jeffery MM et al. *JAMA Network Open.*

eTable 24. Evidence of Tolerance Among Patients Receiving Transdermal Fentanyl Stratified by Initial Dose Strength and Insurance Group

Transdermal Fentanyl	Commercial			Medicare Advantage		
Most common doses mcg/hr	Episodes with evidence of tolerance	Total episodes	Percent Tolerant	Episodes with evidence of tolerance	Total episodes	Percent Tolerant
12	2,236	9,312	24.0%	2,047	13,751	14.9%
25	8,825	26,706	33.1%	5,449	20,915	26.1%
50	4,573	10,522	43.5%	2,486	6,874	36.2%
75	1,367	2,783	49.1%	672	1,572	42.8%
100	889	1,715	51.8%	448	1,005	44.6%
Other doses	159	357	44.5%	100	456	21.9%

© 2020 Jeffery MM et al. *JAMA Network Open.*

Total	18,049	51,395	35.1%	11,202	44,573	25.1%

© 2020 Jeffery MM et al. *JAMA Network Open.*

Comparison to Larochelle, Cocoros, et al. (2) and Willy, Graham, et al. (3)

Demographic comparisons

eTable 25. Distribution of Opioid-Tolerant Only Episodes by Medication Type in Larochelle et al and OptumLabs Data Warehouse

	ER Oxycodone	ER Hydromorphone	Transdermal Fentanyl	Transmucosal Fentanyl	Overall
Sentinel 2009 – 2013	79,824 (44.6%)	7,343 (4.1%)	91,778 (51.3%)	Not included	178,945
OLDW 2007 – 2016	43,559 (28.4%)	5,710 (3.7%)	101,676 (66.3%)	2,440 (1.6%)	153,385

eTable 26. Comparison of Age and Gender in Larochelle et al and OptumLabs Data Warehouse

Sentinel

	Opioid tolerance metric		
	ERO	Extended-release hydromorphone	Transdermal fentanyl
Total (n)	79,824	7,343	91,778
Male	53%	44%	41%
Age			
0-17 years	<1%	<1%	<1%
18-24 years	3%	2%	2%
25-34 years	13%	12%	9%
35-44 years	22%	24%	18%
45-54 years	34%	36%	34%
55-64 years	28%	26%	37%

ERO = extended–release oxycodone

OLDW

	Opioid-Tolerant-Only Episodes				
	Extended-Release Oxycodone	Extended-Release Hydro-morphone	Trans-dermal Fentanyl	Trans-mucosal Fentanyl	Overall
**Total**	30,963	3,198	54,419	1,905	90,485
**Male**	52.5%	43.9%	41.5%	40.9%	45.3%
Age					
0-17	>1.0%	<1.0%	<1.0%	<1.0%	<1.0%
18-24	>2.3%	>1.0%	1.7%	>1.0%	>1.6%
25-34	<11.3%	<11.8%	7.2%	>7.6%	9.0%
35-44	20.6%	22.8%	16.1%	19.4%	17.9%
45-54	31.7%	33.4%	27.8%	32.2%	29.4%
55-64	26.1%	25.5%	29.4%	29.8%	28.1%
65-74	5.6%	4.0%	9.0%	7.0%	7.6%
75+	2.3%	1.0%	8.7%	2.0%	6.1%

© 2020 Jeffery MM et al. *JAMA Network Open.*

eTable 27. Comparison of Age and Gender in Willy et al and OptumLabs Data Warehouse

**Study**	**Willy, Graham, et al. (3)**	**OLDW**	**OLDW**
**Population**	**Medicare FFS**	**Medicare Advantage**	**Medicare Advantage**
	**ER Oxycodone**	**ER Oxycodone**	**All OTO episodes**
**Age category**			
**0-44**	37,726 (13%)	838 (6.7%)	2,315 (3.7%)
**45-64**	105,174 (36%)	6,085 (48.3%)	18,635 (29.6%)
**65-69**	44,654 (15%)	2,025 (16.1%)	9,166 (14.6%)
**70-74**	37,516 (13%)	1,464 (11.6%)	8,905 (14.2%)
**75-79**	28,856 (10%)	1,077 (8.6%)	8,552 (13.6%)
**80+**	38,460 (13%)	1,107 (8.8%)	15,327 (24.4%)
**Male**	122,380 (42%)	5,917 (47.0%)	23,342 (37.1%)

Opioid tolerance rate comparison

eTable 28. Opioid Tolerance Rate by Age in Willy et al vs OptumLabs Data Warehouse

**Study**	**Willy, Graham, et al. (3)**	**OLDW**
**Population**	Medicare FFS	Medicare Advantage
**% opioid tolerant**	ER oxycodone	ER oxycodone
**<65**	47.0%	85.2%
**65-74**	35.5%	85.2%
**75-84**	29.2%	85.2%*
**85+**	21.5%	
* OLDW 75 to 84 category includes a small number of people aged 85+		

eTable 29. Opioid Tolerance Rate by Age in Larochelle et al vs OptumLabs Data Warehouse

**Study**	**Larochelle, Cocoros, et al. (2)**	**OLDW**	**Larochelle, Cocoros, et al. (2)**	**OLDW**	**Larochelle, Cocoros, et al. (2)**	**OLDW**
**Population**	**Commercially insured**	**All**	**Commercially insured**	**All**	**Commercially insured**	**All**
**Episodes**	**ER oxycodone**		**ER hydromorphone**		**Transdermal fentanyl**	
**% opioid tolerant**						

© 2020 Jeffery MM et al. *JAMA Network Open.*

Study	Larochelle, Cocoros, et al. (2)	OLDW	Larochelle, Cocoros, et al. (2)	OLDW	Larochelle, Cocoros, et al. (2)	OLDW
Population	Commercially insured	All	Commercially insured	All	Commercially insured	All
Episodes	ER oxycodone		ER hydromorphone		Transdermal fentanyl	
Age category						
0-17	20.6%	52.9%	44.4%	--	17.7%*	16.9%
18-24	42.2%	58.2%	59.2%	--	38.5%	36.3%
25-34	61.6%	79.1%	63.9%	64.7%	47.9%	46.4%
35-44	65.4%	84.2%	66.3%	66.0%	44.6%	44.1%
45-54	65.4%	85.1%	66.0%	64.1%	41.1%	41.5%
55-64	63.7%	85.4%	60.2%	62.8%	35.3%	36.3%

*The 17.7% opioid tolerance was for the age group 12-17.

eFigure 7. Comparison of Opioid-Tolerant Only Episodes Meeting Tolerance Definitions 1 to 4 in Larochelle et al and OptumLabs Data Warehouse

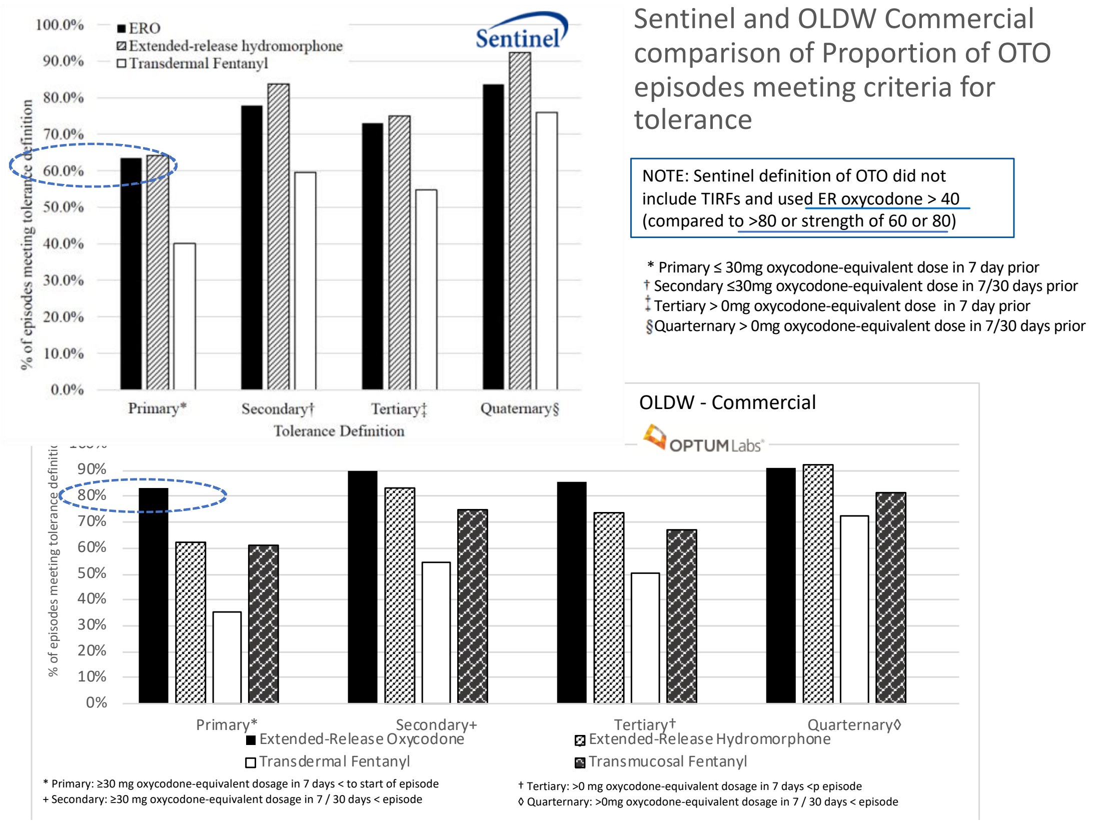

© 2020 Jeffery MM et al. *JAMA Network Open.*

eTable 30. Opioid Poisoning Diagnosis Codes

ICD-9	ICD-10	Code Description
965		Poisoning by opiates and related narcotics
965.00		Poisoning-opium NOS
965.02		Poisoning-methadone
965.09		Poisoning-opiates not elsewhere classified
965.01		Poisoning-heroin
E850.0		Accidental poisoning by heroin
E850.1		Accidental poisoning by methadone
E850.2		Accidental poisoning by other opiates and related narcotics
	T400X4	Poisoning by opium, undetermined
	T400X4A	Poisoning by opium, undetermined, initial encounter
	T400X4D	Poisoning by opium, undetermined, subsequent encounter
	T400X4S	Poisoning by opium, undetermined, sequela
	T400X5	Adverse effect of opium
	T400X1	Poisoning by opium, accidental (unintentional)
	T400X1A	Poisoning by opium, accidental (unintentional), initial encounter
	T400X1D	Poisoning by opium, accidental (unintentional), subsequent encounter
	T400X1S	Poisoning by opium, accidental (unintentional), sequela
	T400X2	Poisoning by opium, intentional self-harm
	T400X2A	Poisoning by opium, intentional self-harm, initial encounter
	T400X2D	Poisoning by opium, intentional self-harm, subsequent encounter
	T400X2S	Poisoning by opium, intentional self-harm, sequela
	T401X4	Poisoning by heroin, undetermined
	T401X4A	Poisoning by heroin, undetermined, initial encounter
	T401X4D	Poisoning by heroin, undetermined, subsequent encounter
	T401X4S	Poisoning by heroin, undetermined, sequela
	T401X2A	Poisoning by heroin, intentional self-harm, initial encounter
	T401X2D	Poisoning by heroin, intentional self-harm, subsequent encounter
	T401X2S	Poisoning by heroin, intentional self-harm, sequela
	T401	Poisoning by and adverse effect of heroin
	T401X	Poisoning by and adverse effect of heroin
	T401X1	Poisoning by heroin, accidental (unintentional)
	T401X1A	Poisoning by heroin, accidental (unintentional), initial encounter
	T401X1D	Poisoning by heroin, accidental (unintentional), subsequent encounter
	T401X1S	Poisoning by heroin, accidental (unintentional), sequela
	T401X2	Poisoning by heroin, intentional self-harm
	T402X1	Poisoning by other opioids, accidental (unintentional)
	T402X1A	Poisoning by other opioids, accidental (unintentional), initial encounter
	T402X1D	Poisoning by other opioids, accidental (unintentional), subsequent encounter
	T402X1S	Poisoning by other opioids, accidental (unintentional), sequela
	T402X2	Poisoning by other opioids, intentional self-harm
	T402X2A	Poisoning by other opioids, intentional self-harm, initial encounter
	T402X2D	Poisoning by other opioids, intentional self-harm, subsequent encounter
	T402X2S	Poisoning by other opioids, intentional self-harm, sequela
	T402X4	Poisoning by other opioids, undetermined
	T402X4A	Poisoning by other opioids, undetermined, initial encounter

© 2020 Jeffery MM et al. *JAMA Network Open.*

ICD-9	ICD-10	Code Description
	T402X4D	Poisoning by other opioids, undetermined, subsequent encounter
	T402X4S	Poisoning by other opioids, undetermined, sequela
	T403	Poisoning by, adverse effect of and underdosing of methadone
	T403X	Poisoning by, adverse effect of and underdosing of methadone
	T403X1	Poisoning by methadone, accidental (unintentional)
	T403X1A	Poisoning by methadone, accidental (unintentional), initial encounter
	T403X1D	Poisoning by methadone, accidental (unintentional), subsequent encounter
	T403X1S	Poisoning by methadone, accidental (unintentional), sequela
	T403X2	Poisoning by methadone, intentional self-harm
	T403X2A	Poisoning by methadone, intentional self-harm, initial encounter
	T403X2D	Poisoning by methadone, intentional self-harm, subsequent encounter
	T403X2S	Poisoning by methadone, intentional self-harm, sequela
	T403X4	Poisoning by methadone, undetermined
	T403X4A	Poisoning by methadone, undetermined, initial encounter
	T403X4D	Poisoning by methadone, undetermined, subsequent encounter
	T403X4S	Poisoning by methadone, undetermined, sequela
	T404X1	Poisoning by other synthetic narcotics, accidental (unintentional)
	T404X1A	Poisoning by other synthetic narcotics, accidental (unintentional), initial encounter
	T404X1D	Poisoning by other synthetic narcotics, accidental (unintentional), subsequent encounter
	T404X1S	Poisoning by other synthetic narcotics, accidental (unintentional), sequela
	T404X2	Poisoning by other synthetic narcotics, intentional self-harm
	T404X2A	Poisoning by other synthetic narcotics, intentional self-harm, initial encounter
	T404X2D	Poisoning by other synthetic narcotics, intentional self-harm, subsequent encounter
	T404X2S	Poisoning by other synthetic narcotics, intentional self-harm, sequela
	T404X4	Poisoning by other synthetic narcotics, undetermined
	T404X4A	Poisoning by other synthetic narcotics, undetermined, initial encounter
	T404X4D	Poisoning by other synthetic narcotics, undetermined, subsequent encounter
	T404X4S	Poisoning by other synthetic narcotics, undetermined, sequela

© 2020 Jeffery MM et al. *JAMA Network Open.*

eReferences

1. National Center for Injury Prevention and Control. CDC compilation of opioid analgesic formulations with morphine milligram equivalent conversion factors, 2015 version. 2015. Available at: http://www.pdmpassist.org/pdf/BJA_performance_measure_aid_MME_conversion.pdf
2. Larochelle MR, Cocoros NM, Popovic J, et al. Opioid tolerance and urine drug testing among initiates of extended-release or long-acting opioids in Food and Drug Administration's Sentinel System. Journal of opioid management 2017;13:315-327
3. Willy ME, Graham DJ, Racoosin JA, et al. Candidate metrics for evaluating the impact of prescriber education on the safe use of extended-release/long-acting (ER/LA) opioid analgesics. Pain medicine (Malden, Mass) 2014;15:1558-1568

© 2020 Jeffery MM et al. *JAMA Network Open.*
